# Pertussis (Whooping Cough)

**DOI:** 10.1093/infdis/jiaa469

**Published:** 2021-09-30

**Authors:** Michael D Decker, Kathryn M Edwards

**Affiliations:** 1 Department of Health Policy, Vanderbilt University Medical Center, Nashville, Tennessee, USA; 2 Department of Pediatrics, Division of Infectious Diseases, Vanderbilt University Medical Center, Nashville, Tennessee, USA

**Keywords:** pertussis, pertussis vaccine, vaccine-preventable diseases, whooping cough

## Abstract

Pertussis (whooping cough) is a respiratory infection caused by *Bordetella pertussis*. All ages are susceptible. In the prevaccine era, almost all children became infected. Pertussis is particularly dangerous in young infants, who account for practically all hospitalizations and deaths, but clinical disease is burdensome at any age. Widespread use of pertussis vaccines dramatically reduced cases, but concern over adverse reactions led to the replacement of standard whole-cell by acellular pertussis vaccines that contain only a few selected pertussis antigens and are far less reactogenic. Routine administration of acellular pertussis vaccines combined with diphtheria and tetanus toxoids is recommended in infancy with toddler and preschool boosters, at age 11, and during pregnancy. Boosting in the second half of every pregancy is critical to protection of the newborn. Waning of vaccine immunity over time has become an increasing concern, and several new pertussis vaccines are being evaluated to address this problem.

Pertussis, commonly known as whooping cough, is a respiratory infection caused by the *Bordetella pertussis* bacteria. Classic pertussis is a cough illness that may last for many weeks and is marked by paroxysms of repeated coughs that end with a gasping “whoop.” Historical records describing a pertussis-like disease go back approximately 1000 years [1], and genetic analyses suggest that the bacterium has been associated with humans for millions of years [2]. Outbreaks of pertussis were recorded in Persia in the 15th century [3], and an outbreak that resulted in many deaths among infants and young children was documented in Paris in the summer of 1578 [4]. British records from the early 16th century and the 1701 London Bills of Mortality reported whooping cough [5]. (Please note that the complete references are available as online Supplemental Materials.)

Pertussis is endemic worldwide, with epidemic peaks every 2–5 years, and is highly contagious. Up to 90% of household contacts and 50% to 80% of schoolroom contacts will become infected after exposure.

## WHY PERTUSSIS IS IMPORTANT

### Infants and Children

Pertussis affects all ages, but most severely infants, who experience the highest age-specific incidence and account for almost all pertussis hospitalizations and deaths. Even now, more than 80% of US infants younger than 2 months with reported pertussis are hospitalized [6].

Pertussis typically begins like a mild upper respiratory infection. An occasional cough progresses within 1 week or 2 to become paroxysmal, increasing in frequency and severity before gradually subsiding over a period of several weeks or longer. Paroxysms are marked by a series of rapid coughs without taking a breath followed by the characteristic whoop, a desperate effort to draw breath through a swollen glottis. During a paroxysm, the patient may become cyanotic and vomiting may follow the paroxysm. Several paroxysms may occur successively within a few minutes, leaving the patient exhausted. Paroxysms may be induced by stimuli such as eating, laughing, or crying, and are typically worse at night. Between paroxysms, the patient appears normal. Pertussis is not usually associated with fever, but it is associated with lymphocytosis, especially among infants and young children. As the illness resolves, nonparoxysmal cough may persist for many weeks and intercurrent viral infections can trigger a recurrence of the paroxysms.

One large study of pertussis in infants and children in Germany, before universal pertussis vaccination, found that 90% of unvacccinated patients with proven pertussis had paroxysmal cough, 79% had whooping, 53% experienced posttussive vomiting, but only 6% had fever [7].

The fearsome reputation of pertussis does not rest merely on its being widespread, prolonged, and highly unpleasant; it can bring permanent disability or death. In the German study, the overall rate of major complications in infants and children was 6%, and it was 24% among infants younger than 6 months [7]. These complications fall into 3 major groups: pulmonary, neurologic, and nutritional. The most frequent are pulmonary complications including both interstitial and alveolar pneumonia and, in severe cases, respiratory failure. Autopsy findings demonstrate lungs filled with inflammatory cells, fibrinous edema, and abundant pertussis bacteria [8]. Pulmonary hypertension can ensue [9–17]; infants younger than 6 weeks of age with pulmonary hypertension have the highest mortality. Patients who survive pneumonia or pulmonary complications typically are left with no permanent lung damage [18].

Neurological complications of pertussis are also reported. Cough paroxysms and associated hypoxia may lead to acute encephalopathy and/or intracranial bleeding. A study in Denmark found that hospitalization for pertussis was associated with an approximately 2-fold higher risk of epilepsy at age 10 years [19]. The US Centers for Disease Control and Prevention has reported that encephalopathy occurs in 0.4% of infants hospitalized with pertussis [20]. Approximately one third of children with pertussis encephalopathy die during the acute illness, and another one third survive with permanent brain damage [21]. The causes of pertussis-related encephalopathy are unclear; possibilities include anoxia due to paroxysmal cough, hypoglycemia, metabolic disturbances, pinpoint intracranial hemorrhages, or a direct toxic effect [22].

Nutritional deficiencies can also arise during pertussis illness, due to the difficulty of feeding in the face of paroxysms of cough and the consequences of posttussive vomiting [23, 24]. Other complications include subconjunctival hemorrhages and epistaxis due to the paroxysms, edema of the face, and ulcers under the tongue. Middle ear infections are also common and are due to the usual otitis-associated pathogens.

### Adolescents and Adults

Pertussis is a burdensome illness in adolescents and adults. A large Canadian study found that adolescents and adults with confirmed pertussis had a median of 8 weeks of cough, including 6 weeks with violent cough; 46% reported vomiting, 84% reported night cough, and 14% reported apnea lasting for 30 seconds after cough [25]. Adults had higher rates of complications than adolescents. A German study of 79 adults with symptomatic pertussis found that 80% had cough > 3 weeks, 63% had prolonged paroxysmal cough, 52% were kept awake by cough, 42% vomited after coughing, but only 8% had whoops; 1 person coughed for 8 months [26]. Attacks of flushing and sweating that lasted 1–2 minutes, occurring several times a day, and continuing for 2 to 8 weeks were reported by 14%. Complications were reported by 23% and included otitis media, pneumonia, urinary incontinence, rib fracture, and severe weight loss. In a Massachusetts study of 314 adolescents and 203 adults with confirmed pertussis, patients reported paroxysmal cough (74% and 84%, respectively), problems sleeping (77% and 84%), vomiting (56% and 54%), urinary incontinence (3% and 28%), weight loss (33% and 33%), rib fracture (1% and 4%), and loss of consciousness (1% and 6%) [27]. Other known complications of pertussis in adults include triggering of migraines [28], carotid artery dissection [29], fainting after cough [30], and loss of memory [31, 32]. Death is rare in adolescents and adults, but it does occur [33–35].

## EPIDEMIOLOGY

Before widespread pertussis vaccination, the United States annually recorded as many as 270 000 pertussis cases with up to 10 000 deaths [36]. However, these only represented the cases that were reported; it is believed that almost every child contracted pertussis. The introduction of pertussis vaccine led to a dramatic decline in reported pertussis.

**Figure 1.**
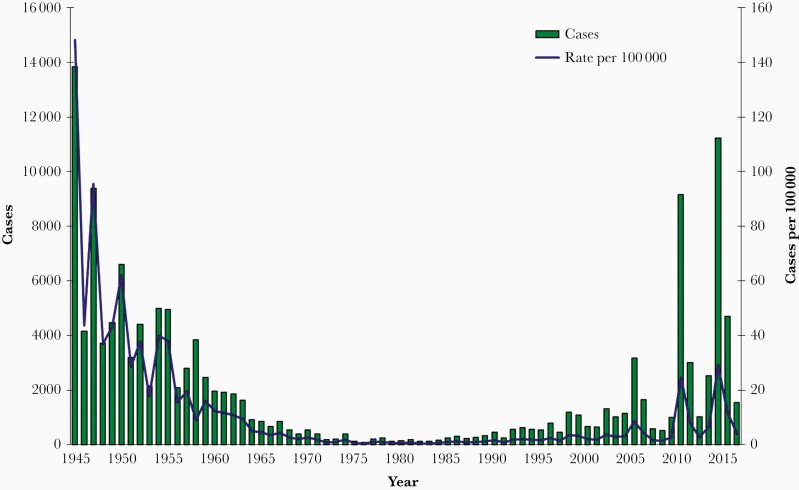
Number and incidence of reported pertussis cases by year of onset—California, 1945–2016 (includes cases reported as of January 23, 2017) (available at https://www.cdph.ca.gov/Programs/CID/DCDC/CDPH%20Document%20Library/Immunization/Pertussis%20report%205-11-2017.pdf).

Although it was once believed that pertussis disease produced lifelong protection, immunity after infection wanes and repeated episodes of illness can occur. Before widespread vaccination, pertussis was so common among children that adolescents and adults repeatedly had their immunity reinforced by a mild or inapparent illness. The curtailment of pediatric pertussis by vaccination meant that immunity among older individuals was no longer being boosted by community exposures and case counts began to increase. During the 1990s, numerous studies in North America, Europe, and Australia demonstrated that pertussis occurred commonly in adults [25, 26, 37–45]. During the late 1990s and early 2000s, pertussis rates in adolescents and adults rose sharply. In the United States, rates in 2000–2003 were 5.5-fold higher for adolescents and 4.9-fold higher for adults than in 1990–1993 [46,^47^,48]. Pertussis rates in adolescents and adults continued to rise until booster vaccination strategies were adopted. As discussed below, maternal vaccination is reducing pertussis among the highest-risk population, young infants. However, as Figure 1 illustrates, baseline pertussis case counts continue to rise, punctuated by outbreaks of a magnitude not seen since the 1940s. Immunological characteristics of the current acellular vaccines contribute to this problem (discussed below), as does the continued presence—indeed, in many locales, growth—of undervaccinated or unvaccinated populations.

Since 1980, the global pertussis case count has fallen more than 90% as immunization coverage has increased [49]. However, assessment is complicated by the fact that pertussis surveillance data tend to be limited or lacking in low- and middle-income countries (LMICs) [50]. Moreover, especially in Africa, marked variations in pertussis vaccine coverage between and within nations still persist, with large areas of coverage below 50% [51]. A serosurvey conducted from 2013 to 2016 in 7 Asian countries found evidence of substantial *B pertussis* circulation, with 1 in 20 subjects having serological evidence of recent infection [52]. A review of pertussis in the 7 countries represented in the Association of Southeast Asian Nations found substantial variation in immunization schedules and pertussis rates, with the true disease burden remaining unclear due to lack of reliable data [53]. Another factor causing considerable variability in the pertussis epidemiology in LMICs is the fact that vaccines incorporating acellular pertussis (aP) components are available in the private market in most countries that use whole-cell pertussis (wP) vaccines in their public vaccination programs, with the relative population penetrations varying substantially from country to country [54].

## BACTERIOLOGY AND PATHOLOGY

*Bordetella pertussis* is a small, pleomorphic, Gram-negative bacillus. The organism contains numerous antigens including pertussis toxin (PT), filamentous hemagglutinin (FHA), pertactin (PRN), several serotypes of fimbrial agglutinogens, adenylate cyclase toxin, tracheal cytotoxin, heat-labile toxin, *Bordetella* resistance to killing protein (BrkA), and endotoxin (Table 1).

**Table 1.** Key Components of the *Bordetella pertussis* Organism

Component	Biological Activity
Pertussis toxin (PT)	A secreted exotoxin that induces lymphocytosis, sensitivity to histamine, and pancreatic islet cell activation. Antibodies to PT are associated with clinical immunity to pertussis.
Filamentous hemagglutinin (FHA)	Involved in attachment to ciliated respiratory epithelium. Mice immunized with FHA are protected against lethal respiratory challenge, and serum antibodies to FHA are found after natural infection and after immunization.
Pertactin (PRN)	An outer-membrane protein that promotes adhesion to ciliated respiratory epithelium. PRN is highly immunogenic. Antibodies to it are found after natural disease and immunization. Mice with antibodies to PRN are highly resistant to an otherwise fatal aerosol challenge with virulent B pertussis.
Fimbriae (FIM)	Involved in attachment to ciliated respiratory epithelium. Antibodies to FIM agglutinate B pertussis and are found almost universally after natural disease or immunization.
Tracheal cytotoxin (TCT)	Induces paralysis and destruction of respiratory ciliated epithelium.
Adenylate cyclase toxin (ACT)	Inhibits phagocytic function. ACT-deficient mutants have reduced ability to cause lethal infection. ACT is not included in any current acellular pertussis vaccines due to protein stability issues.
Heat-labile (dermonecrotic) toxin (HLT)	Causes dermal necrosis and vasoconstriction in animals. It is a weak immunogen and antibodies to it are nonprotective in animal challenge tests.
BrkA	An outer-membrane protein involved in adherence and complement resistance. Similar in structure to PRN. Antibodies to BrkA augment killing of B pertussis.
Endotoxin	Similar to endotoxin in other Gram-negative organisms. Contributes to fever and local reactions.

Since 2007, studies in many countries have identified increasing prevalence of circulating *B pertussis* strains deficient in 1 or more antigens included in aP vaccines [55–66]. This has raised concern that the effectiveness of aP vaccines might be reduced, but such has not been found [67].

*Bordetella pertussis* attaches strongly to ciliated respiratory epithelial cells; important attachment proteins include PT, FHA, fimbrial proteins, PRN, and BrkA [68–73]. The organism generally does not invade submucosal cells or the bloodstream, but its toxins (Table 1) can produce systemic effects. The proteins and toxins of *B pertussis* enable it to interfere broadly with the immune system, including inhibiting complement, phagocytes, and T- and B-cell responses [74, 75].

### Transmission, Carriage, Diagnosis, and Treatment

Pertussis typically is spread through large respiratory droplets generated by coughing or sneezing. However, the baboon model of *B pertussis* infection [76–78] has shown that asymptomatic animals can efficiently spread infection to naive baboons housed separately but nearby, and silent transmission has been observed in humans [79]. The advent of sensitive polymerase chain reaction (PCR) assays has shown that nasopharyngeal samples from asymptomatic vaccinated persons exposed to pertussis cases are often PCR-positive [80–84], and that there is a high prevalence of asymptomatic infection among household-exposed persons [85].

Culture was traditionally the gold standard for pertussis diagnosis, but it has substantial limitations. The organism is fastidious and requires special transport and culture techniques. Moreover, the ability to recover organisms in culture fades early in illness, often before pertusssis is suspected [86–90]. In contrast, PCR can permit detection several weeks into the illness and nonviable organisms can be detected. Serology is still commonly used, particularly in clinical trials and for epidemiological purposes [39, 91–100]. Serological diagnosis is most reliable when based on a significant antibody rise from acute to convalescent samples, but often only a convalescent specimen is available, in which case the result can be compared with a prespecified threshold [38, 40, 91, 101–106].

The administration of macrolides early in illness reduces illness duration and severity and lessens infectivity. Postexposure prophylaxis reduces the risk of pertussis [107–108] by eradicating *B pertussis* from the nasopharynx [36, 109–119]. Macrolide-resistant strains have become increasingly prevalent in China and have appeared occasionally elsewhere [120]. Although most untreated persons spontaneously clear *B pertussis* in 3–4 weeks after cough onset, the organism can persist for 6 weeks or more [121, 122]. Cases and inadequately vaccinated contacts should be excluded from school, day care, and similar settings until they have received at least 5 days of treatment [123].

## WHOLE-CELL PERTUSSIS VACCINES

By the late 1940s, pertussis vaccines made from suspensions of killed organisms (called whole-cell vaccines) had been proven effective in clinical trials and were available in combination with diphtheria and tetanus toxoids (DTP). These DTP vaccines were routinely used in the United States and many other countries, leading to dramatic declines in pertussis morbidity and mortality. These vaccines currently remain in use in most LMICs.

### Benefits

The effectiveness of wP vaccines has been demonstrated repeatedly, beginning with studies in the Faroe Islands in 1923 and 1929 [124] and in Michigan in the early 1930s [125]. During the studies of diphtheria, tetanus, and aP (DTaP) vaccines conducted in the 1980s in Germany [126, 127], England [128], Sweden [129], Italy [130], and Senegal [131], 5 DTP comparator vaccines were evaluated, and their efficacies against protocol-defined symptomatic illness ranged from 83% to 98% for 4 of the vaccines. One US vaccine was substantially less efficacious (36% to 48%) [129, 130].

Additional proof of the effectiveness of pertussis vaccines was provided by 3 countries that initially adopted, but then reduced or eliminated, pertussis vaccination. Japan introduced pertussis vaccine in 1949, and reported pertussis cases and deaths declined markedly [132, 133]. However, in 1975, widely publicized concerns about adverse events alleged to be due to DTP vaccine led to the cessation of infant-toddler pertussis vaccination [134]. Epidemic pertussis recurred; cases rose from 206 in 1971 to over 13 000 in 1979, with hundreds of childhood deaths [132, 134]. A similar pattern was seen in England and Wales, where pertussis vaccine acceptance rates fell to 25% during the mid 1970s. Major epidemics of pertussis returned, with numerous deaths [135, 136]. In 1979, Sweden suspended the use of a DTP vaccine that was considered poorly effective; the incidence of whooping cough more than quadrupled thereafter, with several major outbreaks [137, 138]. In each country, after eventual resumption of pertussis vaccination (using whole-cell vaccine in the UK, acellular vaccine in Japan and Sweden), pertussis rates again declined [134, 136, 138, 139].

The benefit of pertussis vaccination is not measured solely by disease avoided but also by disease ameliorated. Particularly during outbreaks, the pressure of infection is sufficiently high that properly vaccinated persons may nonetheless contract pertussis disease. Multiple studies have demonstrated that such breakthrough cases are substantially milder than cases in unvaccinated or undervaccinated persons [135, 140–145].

### Adverse Events With Whole-Cell Vaccines

Whole-cell pertussis vaccines are among the most reactogenic of the routinely administered vaccines. Minor local (injection-site redness, swelling, and pain) and systemic (fever, irritability, and drowsiness) reactions occur in approximately half of DTP recipients—5 times the rate seen with diphtheria and tetanus (DT) vaccine [146–149]. Unusual high-pitched crying or persistent inconsolable crying beginning 2–8 hours after vaccination and lasting 1 hour or more is reported to occur in 3% to 4% of children [149, 150]. Diphtheria and tetanus toxoids is also associated, uncommonly, with febrile seizures and hypotonic-hyporesponsive episodes (HHE), shock-like states that begin within 12 hours of vaccination, last for several hours, and resolve without sequelae. Febrile seizures and HHE both occur in approximately 0.06% of vaccinated children [149, 151].

The most serious reaction that was attributed to DTP was acute encephalopathy. The identification of Dravet Syndrome, an epileptic encephalopathy due to a sodium-channel mutation [152], has broadened the understanding of encephalopathy after vaccination [153]. It is believed that the fever associated with DTP likely unmasked the syndrome and consequent seizures in such children, rather than the vaccine itself causing encephalopathy.

Concerns regarding DTP grew in the early 1970s, particularly in countries where widespread vaccination had eliminated most disease and young parents and physicians had never witnessed the morbidity and mortality of whooping cough. Although school-entry immunization laws enabled US vaccination rates to be maintained, litigation alleging vaccine injury contributed to the discontinuation of pertussis vaccine production by most US manufacturers. This prompted the US Congress to pass the National Childhood Vaccine Injury Act, which limits civil suits while providing rapid and certain compensation for adverse events recognized to be vaccine-related.

During the early 1990s, the Institute of Medicine (IOM) carefully reviewed the evidence for a causal relation between vaccines and various adverse events [154, 155–157]. The IOM concluded that no causal relationship existed between DTP vaccination and sudden infant death syndrome, afebrile seizures, infantile spasms, or Reye syndrome. In contrast, the evidence was deemed to indicate DTP being a cause of anaphylaxis, prolonged or inconsolable crying, and febrile seizures and to be consistent with causation of acute encephalopathy and HHE.

## PEDIATRIC ACELLULAR PERTUSSIS VACCINES

Efforts to develop less reactogenic pertussis vaccines began in Japan after their cessation of DTP vaccination in the mid-1970s. The initial aP vaccines were composed predominantly of FHA along with smaller amounts of inactivated PT and, often, traces of fimbrial proteins and PRN [134]. Other aP vaccines containing equal quantities of PT and FHA soon followed. Acellular pertussis vaccines have been used exclusively in Japan since 1981; household-contact studies and pertussis surveillance after implementation of the acellular vaccines demonstrated their effectiveness [134, 158–165].

During the 1980s, some 2 dozen acellular vaccines were developed in various countries and demonstrated much lower rates and severities of adverse reactions. Diphtheria, tetanus, and aP vaccines have entirely replaced DTP in the United States, Canada, Australia, most of Europe, and in some Asian and Latin American countries and are available in private markets in almost every country.

### Multicenter Acellular Pertussis Trial

In the early 1990s, the US National Institutes of Health (NIH)-sponsored Multicenter Acellular Pertussis Trial (MAPT) evaluated 13 acellular and 2 whole-cell vaccines head-to-head to determine the most promising for inclusion in subsequent large-scale efficacy trials [166]. The DTaP vaccines differed with respect to the number of included *B pertussis* proteins, the amount of each included protein, the methods of manufacture and detoxification of the proteins, and the choice of adjuvants and excipients [167]. Antibody responses differed substantially amongst the acellular vaccines, but each vaccine stimulated significant antibody responses to its included antigens, in most cases equaling or exceeding the antibody response produced by the reference whole-cell vaccine [167].

### Efficacy Trials

Between 1985 and 1993, 9 large studies of aP vaccines were conducted in Europe or Africa in areas not routinely vaccinating against pertussis. The trial protocols and included vaccines differed substantially [168]. Nonetheless, despite their limitations and age, these studies remain among the best sources of information regarding the evaluated vaccines. New pertussis efficacy trials are unlikely; pertussis vaccines are globally recommended, making a placebo arm not possible, and current vaccines are so effective in the first few years of life that the sample size of a comparative trial would be prohibitive.

A study conducted in 1986 in Stockholm [138, 169, 170] compared 2 Japanese vaccines, one containing both PT and FHA and the other only PT, but in larger quantity. Initial efficacy results were disappointing at 69% and 54%, respectively, but later analyses using a case definition of culture-confirmed disease with at least 21 days of spasmodic cough (a definition adapted by the World Health Organization [WHO] and used in subsequent efficacy trials) found efficacies of 81% and 75%, respectively [171]. The vaccine containing more PT was more effective in preventing severe manifestations of pertussis, whereas the vaccine containing both PT and the attachment protein FHA was more effective in preventing mild or moderate disease [138, 172, 173].

In the early 1990s, various candidate DTaP vaccines were evaluated in 8 efficacy trials, of which 4 were sponsored by the NIH and 4 by the manufacturers of the DTaP evaluated. All of the NIH-sponsored studies were prospective, double-blinded, randomized, and contained a whole-cell control arm, a placebo group, or both. A study based in Göteborg, Sweden (1991–1994) vaccinated children at 3, 5, and 12 months (the standard Swedish schedule) with Certiva, a DTaP vaccine containing 40 mcg of peroxide-detoxified PT; no comparison vaccine was included [174]. (Here and below, brand names are used because DTaP vaccines all have similar or identical generic names.) Based on the WHO case definition, the efficacy of Certiva was 71%. A study conducted in Italy (1992–1993) compared a Connaught US DTP against Infanrix and Acelluvax, each of which contained PT, FHA, and PRN (see Table 2 footnotes for details) given at 2, 4, and 6 months. The acellular vaccines were both 84% effective, whereas efficacy for the Connaught whole-cell vaccine was only 36% [130]. A companion study in Stockholm (1992–1993) evaluated Tripacel and a 2-component DTaP (never licensed) using the same schedule and DTP control; efficacies were 85% for Tripacel and 48% for the DTP. A second study in Stockholm (1993–1996) evaluated the higher-potency formulation of Tripacel used in subsequent combination vaccines (see Table 2) against Acelluvax, using a British DTP (Medeva-Wellcome) as control. The relative risk of WHO-defined pertussis was 38% lower for the higher-potency Tripacel compared with Acelluvax (because there was no placebo group, absolute efficacy cannot be calculated) [129].

**Table 2.** Selected Solicited Adverse Events Occuring by the Third Evening After Vaccination (at 2, 4, or 6 Months of Age) in the Multicenter Acellular Pertussis Trial [150] and Selected Subsequent Efficacy Trials [129, 130, 131, 175]

	Multicenter Acellular Pertussis Trial							Efficacy Trials		
Vaccine	Temperature >38.4°C	Redness >20 mm	Swelling >20 mm	Pain^a^	Fussiness^b^	Drowsiness	Anorexia	Crying ≥3 Hours	HHE	Seizures 1st 48 Hours
Whole-cell^c^	15.9%	16.4%	22.4%	40.2%	41.5%	62.0%	35.0%	11.5%	1.7%	0.06%
Acelluvax^d^	1.6%	1.6%	2.4%	1.6%	16.7%	41.3%	19.0%	1.3%	0%	0%
Infanrix^e^	3.3%	4.2%	5.8%	10.8%	15.0%	46.7%	19.2%	1.9%	0.2%	0.002%
Triavax^f^	4.6%	4.5%	5.3%	8.3%	12.0%	42.1%	20.3%	n/a	n/a	0.09%
Tripacel^f^	3.6%	3.6%	4.4%	5.1%	18.2%	42.3%	19.0%	n/a	0.04%	0%

Abbreviations: HHE, hypotonic hyporesponsive episode; n/a, not applicable.

^a^Moderate (cried or protested to touch) or severe (cried when leg moved).

^b^Moderate (prolonged crying and refused to play) or severe (persistent crying and could not be comforted).

^c^Whole-cell vaccines: MAPT, manufactured by Wyeth-Lederle Vaccines and Pediatrics, USA, no longer available; efficacy trials: manufactured by Connaught Laboratories, USA, no longer available.

^d^A 3-component vaccine (also known as Triacelluvax) manufactured by Chiron Vaccines that contained 5 mcg of genetically inactivated pertussis toxin (PT) and 2.5 mcg each of filamentous hemagglutinin (FHA) and pertactin (PRN). No longer available.

^e^A 3-component vaccine manufactured by GlaxoSmithKline, Belgium that contains 25 mcg each of PT and FHA and 8 mcg of PRN. Also available as a component in Boostrix, Infanrix-IPV, Infanrix hexa, Kinrix, Pediarix, etc.

^f^A 2-component vaccine manufactured by Sanofi Pasteur, France that contains 25 mcg each of PT and FHA (also known as Triaxim). Not available except as a component in Hexaxim, Hexacima, Hexyon, Tetraxim, Pentaxim, etc.

^g^A 5-component vaccine manufactured by Sanofi Pasteur, Canada. Also available as Daptacel (contains 10 mcg PT, 5 mcg FHA, 3 mcg PRN, and 5 mcg of fimbriae) and (with the PT and FHA increased to 20 mcg each) as a component in Pentacel, PediaceI, Quadracel, Adacel, Adacel-Polio, and in MCM Vaccine Company’s Vaxelis.

Of the 4 manufacturer-sponsored efficacy trials, only 1 evaluated a vaccine that remains globally available. A study in Senegal (1990–1994) compared Triavax with that company’s French-manufactured DTP vaccine [131]. Estimates of absolute efficacy in this randomized, double-blind study were derived from a nested case-contact study that compared rates of pertussis after exposure to an index case among study subjects vaccinated at 2, 4, and 6 months of age and nonstudy children (who received either DT or no vaccine) living in the same housing compounds. The absolute efficacy estimates were 74% (95% confidence interval [CI], 51%–86%) for Triavax and 92% (95% CI, 81%–97%) for DTwP; the confidence limits are wide because the total number of cases was small, particularly in the group that had not received pertussis vaccine. Additional manufacturer-sponsored efficacy trials were conducted in (1) Munich, 1993–1995 [127, 176], (2) Erlangen, Germany, 1991–1994 [177], and (3) Mainz, Germany, from 1992–1994 [126]. Interested readers are referred to their respective reports for details.

### Conclusions From the Efficacy Trials and Follow-up Studies

The studied aP vaccines were associated with markedly lower rates of adverse events compared with whole-cell vaccine, while providing effectiveness that approached or exceeded that of whole-cell vaccine. Although the vaccines differed in efficacy, national surveillance data have shown that each acellular vaccine adopted for routine use has provided excellent control of pertussis during the ages covered by the childhood vaccination series [59, 178–183]. The first dose of aP vaccine provides modest but important protection [184]; the second dose, provides substantially more protection [168, 185]. The best protection is achieved with 3 primary doses (whether at 2, 4, and 6 months; 3, 5, and 7 months; or even 2, 3, and 4 months) with a booster in the second year of life [179, 180, 182]. The Scandinavian schedule (3, 5, and 12 months) gives a little less protection between the second and third doses but better protection thereafter, compared with the other 3-dose schedules.

### Adverse Events With Pediatric Acellular Vaccines

Many studies comparing acellular to whole-cell vaccines have been conducted in infants and children [186–193] and have invariably found the acellular vaccines to be associated with lower rates of adverse reactions than whole-cell vaccine. Table 2 shows rates of solicited adverse reactions observed in the MAPT and selected efficacy trials for the 3 aP vaccines that remain commercially available in 2020, their control whole-cell vaccines, and the only evaluated DTaP vaccine containing a genetically inactivated PT component.

#### Safety of Booster Doses

Follow-up studies to the MAPT evaluated fourth and fifth doses of acellular vaccine given at ages 15–20 months and 4–6 years, respectively [194, 195]. Injection site redness, swelling, pain, and fever were seen more frequently with the fourth consecutive dose of acellular vaccine than with the primary series, but still at rates lower than seen in children given 4 consecutive doses of whole-cell vaccine. Several cases of swelling of the entire limb were observed after administration of the fourth dose of acellular vaccine. Of 1015 children given 4 successive doses of aP vaccine, 20 (2%) reported swelling of the entire thigh after the fourth dose versus none of 246 children primed with whole-cell and boosted with acellular vaccine [196]. Among children given 5 successive doses of acellular vaccine, 4 (1.5%) of 267 experienced swelling of the whole upper arm after the fifth dose (*P* = .13). Swelling began on day 1–2 after vaccination and generally resolved by day 4, with no sequelae [196]. The occurrence of whole-limb swelling after the fourth or fifth consecutive dose of aP vaccine has been noted in many other studies [197–204]. In a study of 20 children who experienced large limb swelling after a fourth dose of acellular vaccine, only 4 had swelling of the entire upper arm after a fifth dose [204]. Most commonly, whole-limb swelling is not accompanied by pain, redness, or distress and resolves without sequelae in a few days; it is not known to be related to limb(s) used for prior vaccinations. Rather, there is evidence suggesting that the likelihood of this reaction is related to prevaccination antibody levels in the child and the quantity of antigen in the vaccine [196, 198, 202, 203]. Although whole-limb swelling was considered novel when first described after acellular vaccines, an analysis of VAERS (Vaccine Adverse Event Reporting System) reports published in 2003 found that large local limb swelling was reported equally often after acellular or whole-cell vaccine [205].

In summary, multiple studies have found that adverse reactions occur less frequently—typically, substantially less frequently—after acellular compared with wP vaccines. Reaction rates increase successively with each acellular booster dose but remain lower than seen among children primed and boosted with whole-cell vaccine. Of course, adverse events that occur after vaccination but that are not actually caused by vaccination will continue to occur at their background rates regardless of the vaccine used.

## ADOLESCENT-ADULT ACELLULAR PERTUSSIS VACCINES

The vastly improved tolerability of aP vaccines, compared with whole-cell vaccines, opened the door to extending pertussis vaccination beyond childhood. Numerous aP vaccines have been evaluated in adolescents and adults and have been shown to be safe and immunogenic [146, 206–211].

In recognition of their lower diphtheria and pertussis antigen content, adolescent-adult acellular formulations are designated as tetanus, diphtheria, and pertussis (Tdap) vaccines. The Tdap vaccines developed by GlaxoSmithKline and Sanofi Pasteur are available worldwide, and additional Tdap vaccines have recently been licensed in Asia (Bionet-Asia) or are under development (Serum Institute of India).

An NIH-sponsored study found the the pertussis components of 1 Tdap vaccine to be 92% (95% CI, 32%–99%) efficacious in preventing pertussis, which meets the primary case definition [212]. The aP vaccine was well tolerated; fewer than 5% of subjects reported local or systemic adverse reactions. The study and control groups did not differ in the rate of severe adverse events. Likewise, a study during a pertussis outbreak among Australian high-school students found an effectiveness of 85% (95% CI, 83%–88%) for laboratory-confirmed cases [213].

In 2005, shortly after US licensure of Tdap vaccines, the US Advisory Committee on Immunization Practices (ACIP) recommended a dose of Tdap in lieu of tetanus and diphtheria (Td) vaccine for all adolescents (preferably, at age 11–12 years) and adults. Subsequent studies demonstrated the beneficial impact of this Tdap vaccine on the risk or rates of pertussis among adolescents [214–218]. Current recommendations are discussed below.

### Vaccination in Pregnancy

Despite the success of the childhood pertussis vaccination program and the introduction of adolescent Tdap vaccination, pertussis deaths among infants 0–3 months of age continued to rise, from a total of 49 deaths during the years 1980–1989 to 84 during 1990–1999 to 175 during 2000–2009 [219]. The use of Tdap to create a “cocoon of protection” around newborns by vaccinating expectant or new mothers and other close contacts (father, siblings, grandparents, etc) was shown to be effective when well implemented [220] but was very difficult to implement well [221–224]. These difficulties stimulated interest in the use of Tdap during pregnancy, but 2 concerns had to be addressed: safety (both for mother and fetus) and potential suppression of responses to infant pertussis vaccination (due to elevated transplacental antibody levels). After initial studies assuaged both concerns [225, 226], the ACIP in 2011 recommended administration of Tdap during pregnancy. Numerous subsequent studies have demonstrated the safety and effectiveness of Tdap vaccination during pregnancy [225–229, 230, 231–242]. The Tdap vaccination during pregnancy provides the newborn with high levels of transplacentally derived maternal antibody, which provides protection during the period of greatest risk, before the infant vaccination series begins. The increased transplacental antibody moderately reduces the infant’s antibody responses to the first and second infant DTaP doses, but antibody levels after the third and fourth doses are little affected [225, 229, 243, 244]. Given that the first 3 months of life are by far those of highest risk of pertussis morbidity and mortality, this is a highly beneficial tradeoff, as demonstrated by recent declines in pertussis among infants 2 months of age and younger [238]. Because of rapid post-Tdap waning of pertussis antibody levels, ACIP recommended in 2012 that women receive Tdap during every pregnancy, regardless of timing of prior vaccinations. Similar recommendations have been adopted by many countries; for example, as of 2019, at least 14 countries in the Americas recommend intrapartum Tdap, as do the United Kingdom, Ireland, Italy, Germany, Switzerland, Portugal, Spain, Belgium, New Zealand, and Australia, among others [245, 246].

### Adverse Reactions in Adolescents and Adults

Large clinical trials of the Tdap vaccines among adolescents and adults primed with whole-cell vaccine showed that the addition of purified aP components to Td added essentially nothing to the adverse reaction profile [247–249]. Several very large safety studies of Tdap in adolescents and adults found no evidence of important adverse events (including neurologic reactions, allergic reactions, or new-onset chronic illnesses) [250–252]. Studies in adolescents and adults have not found that adverse reactions to Tdap were significantly increased by recency of prior pertussis vaccination [250, 253].

## DURATION OF PROTECTION FOR ACELLULAR PERTUSSIS VACCINES

Since 2010, multiple large pertussis outbreaks have demonstrated increasing rates of pertussis in adolescents and older schoolchildren correlated with the transition from those whose primary vaccination was entirely with whole-cell vaccine, to those who initially received whole-cell vaccine and subsequently acellular vaccine, to those who received only acellular vaccine [214–218, 221, 254, 259, 263]. Studies have also documented an increase in pertussis activity beginning at 7 years of age among cohorts that received only acellular vaccines and that the effectiveness of the adolescent Tdap booster has waned more quickly among acellular-only recipients than among older cohorts who were primed with 1 or more doses of wP vaccine [213, 217, 218, 254–256, 264–273]. Klein et al [263] reported in 2012 that after the fifth dose of aP vaccine, the odds of acquiring pertussis rose an average of 42% per year. In 2019, Zerbo et al [274] reported that among aP-vaccinated children ages 19–84 months, the risk of pertussis was 5 times higher 3 years versus 1 year after last vaccination. Among vaccinated children 7–11 years of age, risk was twice as high 6 years versus 3 years after last vaccination. A comparison of the 2010 and 2014 California epidemics (Figure 1) found an increasing role of older adolescents in outbreak propagation [275].

Animal models and in vitro studies have provided substantial data that likely explain this phenomenon. Mouse models of *B pertussis* infection demonstrate strong Th1 and Th17 T-helper cell responses after natural infection or whole-cell vaccination, whereas acellular vaccines stimulate a mixed Th2 and Th17 response, with the Th2 component not appearing to materially improve protection [276, 277]. It has been shown that there are persistent differences in T-cell polarization and proliferation in persons originally primed with aP, compared with wP, despite repeated aP boosters [278]. Unfortunately, it appears that the type of pertussis vaccine with which an infant is primed determines T-cell polarization [279]; there is no evidence thus far that subsequent exposure to wP or *B pertussis* can change the immune response pattern from Th2 to Th1 dominance.

Memory cells resident in the nasopharynx and lung seem to be important mediators of protection in the mouse, and they are stimulated much more robustly by natural infection and whole-cell vaccine than by acellular vaccine [280, 281]. Likewise, memory T cells resident in the nasopharynx stimulate production of secretory immunoglobulin (Ig)A that provides protection against *B pertussis* nasal colonization [282].

The baboon model of pertussis has been important in validating findings in the mouse model and further advancing our understanding of pertussis infection and vaccine protection [76–78, 283]. Baboons infected with *B pertussis* manifest all the findings of human pertussis. In the baboon, pertussis infection stimulates strong Th1 and Th17 responses and confers sterilizing immunity. Baboons vaccinated with wP have similar, but less robust, responses. Baboons vaccinated with aP mount strong Th2, low Th1, and no Th17 responses. When aP-vaccinated baboons are challenged with *B pertussis*, they have no clinical symptoms but develop persistent heavy nasopharyngeal colonization, whereas those challenged after wP vaccination are colonized less heavily and clear the organism more rapidly. Baboons vaccinated with aP who become colonized can transmit *B pertussis* to nearby unprotected baboons [283]. These findings in the mouse and baboon models are consistent with the observation that pertussis outbreaks are increasing in frequency and size in aP-vaccinated populations.

A case-control study conducted during an outbreak in California in 2010 found that receipt of all 5 recommended doses of aP vaccine was 89% (95% CI, 79%–94%) effective overall; efficacy declined from 98% within 1 year to 71% 5 or more years later [284]. A similar study conducted during the 2012 Washington State outbreak found that Tdap efficacy among adolescents was 73% within 1 year of vaccination but declined to 34% 2–4 years postvaccination [217]. Likewise, data from the 2012 Wisconsin outbreak showed that children ages 11–12 years played the largest role in propagating the outbreak [285].

## CHOICE OF VACCINES

Multicomponent combination vaccines are typically used for childhood vaccination programs worldwide. Public vaccination programs in Europe, North America, Australia, New Zealand, and parts of Asia and Latin America use vaccines incorporating aP components; manufacturers include GlaxoSmithKline, MCM (Merck-Connaught-Merieux) Vaccine Company, and Sanofi. Some jurisdictions allow the provider to choose from available alternative products; others specify a single product on a tender basis. The rest of the world uses multicomponent combination vaccines containing wP components, typically on a tender basis; manufacturers are almost entirely in Asia and include Biological E, LG Chemical, Panacea Biotech, PT Bio Farma, and Serum Institute of India.

## RECOMMENDATIONS

Pertussis vaccine is recommended worldwide for infants and young children and recommended in many countries for adolescents, adults, pregnant women, and others; details are available at WHO websites [245, 286, 287].

In the United States, the ACIP recommends that every infant receive pertussis vaccine at ages 2, 4, and 6 months, 15 to 18 months, and 4–6 years [288]. The Tdap vaccine is recommended at least once for every person age 11 or older; periodic Tdap boosters have been considered but not recommended due to rapid pertussis antibody waning and consequent poor cost-utility. However, ACIP has recently recommended that Tdap can be used whenever Td would be indicated, thus easing provider inventory management and permitting elective decennial Tdap boosting. More importantly, Tdap is also recommended during weeks 27–36 of every pregnancy. In 2016, the United Kingdom modified their maternal Tdap recommendation to include weeks 20–32 of pregancy; this has reduced the incidence of pertussis among premature infants, without adverse effect on protection of full-term infants [289]. Pertussis vaccine is contraindicated in persons with a history of anaphylaxis or encephalopathy after prior pertussis vaccine.

Recommendations are beneficial only if followed. Although antivaccine sentiments have circulated since the time of Jenner [290–291], the global increase in vaccine hesitancy or outright antivaccine activism in recent decades has proved challenging. Despite overwhelming scientific evidence that vaccines prevent far more mortality and morbidity than they cause, when people cannot personally see the benefits of vaccination (illnesses that did not happen, deaths that did not occur), they may see only risks, often exaggerated or invented [291]. Numerous studies have shown that avoidance of vaccination—typically, through personal belief or unwarranted medical exemptions from school-entry vaccination requirements—results in increased risk of disease, not merely for the un(under)-vaccinated child but also for persons whom that child exposes, persons who may have medical contraindications to vaccination, immune deficits that elevate their risk despite vaccination, or simply breakthrough disease despite vaccination [292–295].

## FUTURE VACCINES

The inability of current acellular vaccines to stimulate the same kinds of cellular immune responses seen with whole-cell vaccines or natural disease, and the apparent consequences of that difference with respect to durability of immunity and protection from carriage and transmission, have stimulated many efforts to develop new pertussis vaccines that preserve the safety of current acellular vaccines while improving their cellular immune response. Although some have called for a return to traditional whole-cell vaccines, that clearly would not be acceptable to those countries that previously had deep concerns about whole-cell vaccine adverse reactions.

Vaccine development efforts have mostly fallen into 3 categories: modification of *B pertussis* for use in whole-cell vaccines with decreased potential for adverse reactions; enhancement of aP vaccines by inclusion of additional or different *B pertussis* antigens, seeking wP-like immunological responses while retaining aP-like safety; and enhancement of aP vaccines by adding an adjuvant that drives immune responses toward the pattern seen with wP vaccines. The exemplar of the first approach has been the development of the BPZE1 *B pertussis* variant, constructed by the genetic inactivation or removal of 3 major toxins [296–308]. BPZE1 is administered intranasally and has been shown to protect against lung and nasal *B pertussis* colonization both in mice and baboons. A clinical trial in human volunteers demonstrated the vaccine’s safety and ability to induce colonization in more than 80% of recipients, with seroconversion in 100% of those given the highest dose of the vaccine [309]. All vaccinees had high blood IgG and IgA titers at day 28, and specific memory B cells were detected. The antigen specificity of these antibodies, characterized by 2-dimensional immunoblotting, was broader compared with the antibody repertoire from aP-vaccinated subjects and was predominantly directed to antigens not present in the aP vaccines. Further studies with this vaccine are underway.

A number of investigators have pursued the second approach of enhancing aP vaccines by adding new or different *B pertussis* antigens [310, 311–322]. Several investigators have evaluated *B pertussis* outer membrane vesicles (OMVs) [317, 318, 322, 323], which contain multiple antigens including secreted and membrane-bound virulence factors. An OMV vaccine developed by Hozbor et al [322] induced immunity in mice that was superior to that of aP and comparable to that of wP. Also under investigation is the incorporation of new or additional antigens including biofilm-associated virulence factors [319, 324], iron uptake proteins [314, 315], adenylate cyclase [315, 325] or other proteins [320], as well as treatment with monoclonal antibodies [326].

The third alternative being explored involves the addition of novel adjuvants to elicit a more Th1-skewed response [304, 312, 327–330]. For example, the Mills group [330] has shown that addition of a Toll-like receptor (TLR)-7 agonist to aP vaccine enhanced Th1 and Th17 responses in mice, yielding protection after challenge comparable to that of wP vaccines. They have also developed a novel adjuvant that combines an interferon stimulator with a TLR-2 agonist and shown its ability to stimulate Th1 and Th17 responses and intranasal tissue-resident T-cells [304]. Other adjuvants under consideration include *Bordetella* colonization factor A [331], CpG TLR-9 agonists [328], and lipopolysaccharide [320].

## CONCLUSIONS

In the absence of systematic vaccination with pertussis vaccine, essentially every person will suffer whooping cough during infancy or childhood; many will suffer serious sequelae or die. No medical intervention is risk-free, but modern pertussis vaccines are both effective and safe—indeed, vastly safer than avoiding vaccination. Pertussis is an endemic and highly contagious disease; outbreaks will continue to occur. In an outbreak, vaccinated children are less likely to be infected; if infected, illness will be less severe [51–53, 332]. Unvaccinated children are at much higher risk of infection and, if infected, at much higher risk of adverse outcome. A number of approaches to new vaccines with more durable protection are under investigation.

## Supplementary Data

Supplementary materials are available at *The Journal of Infectious Diseases* online. Consisting of data provided by the authors to benefit the reader, the posted materials are not copyedited and are the sole responsibility of the authors, so questions or comments should be addressed to the corresponding author.

The complete references are available as online Supplemental Material.

jiaa469_suppl_Supplementary-Material

## Notes

***Supplement sponsorship.*** This supplement is sponsored by the Bill and Melinda Gates Foundation.

***Potential conflicts of interest.*** M. D. D. has received salary, consulting fees, and retirement benefits from Sanofi Pasteur and consulting fees from GlaxoSmithKline. K. M. E. has received consulting fees from Merck, BioNet, and IBM, and serves on Data Safety and Monitoring Committees for the National Institutes of Health, Sanofi, X-4 Pharma, Seqirus, Moderna, and Pfizer. All authors have submitted the ICMJE Form for Disclosure of Potential Conflicts of Interest. Conflicts that the editors consider relevant to the content of the manuscript have been disclosed.
