# Supplementary material for: Pertussis (Whooping Cough)
Source: J Infect Dis. 2021 Sep 30;224(Suppl 4):S310–20. doi: 10.1093/infdis/jiaa469 (PMC8482022; doi:10.1093/infdis/jiaa469)
Supplement: jiaa469_suppl_Supplementary-Material [file jiaa469_suppl_supplementary-material.pdf]

References

1 Weston R. Whooping cough: a brief history to the 19th century. Can Bull Med Hist 2012;29:329-349. doi: 10.3138/cbmh.29.2.329.

2 Diavatopoulos DA, Cummings CA, Schouls LM, Brinig MM, Relman DA, Mooi FR. Bordetella pertussis, the causative agent of whooping cough, evolved from a distinct, human-associated lineage of B. bronchiseptica. PLoS Pathog 2005;1:e45. Epub 2005 Dec 30.

3 Aslanabadi A, Ghabili K, Shad K, Khalili M, Sajadi MM. Emergence of whooping cough: notes from three early epidemics in Persia. Lancet Infect Dis 2015;15:1480-4. doi: 10.1016/S1473-3099(15)00292-3. Epub 2015 Aug 19.

4 Holmes WH: Bacillary and Rickettsial Infections, Acute and Chronic: A Textbook: Black Death to White Plague. New York, Macmillan, 1940.

5 Hardy A. Whooping cough. In Kiple KF (ed): The Cambridge World History of Human Disease. New York, Cambridge University Press, 1993. pp 1094–1096.

6 Clark TA. Changing pertussis epidemiology: everything old is new again. J Infect Dis 2014;209:978-981.

7 Heininger U, Klich K, Stehr K, et al. Clinical findings in Bordetella pertussis infections: results of a prospective multicenter surveillance study. Pediatrics 1997;100:E10.

8 Paddock CD, Sanden GN, Cherry JD, et al. Pathology and pathogenesis of fatal Bordetella pertussis infection in infants. Clin Infect Dis 2008;47:328–338.

9 Halasa NB, Barr FE, Johnson JE, et al. Fatal pulmonary hypertension associated with pertussis in infants: does extracorporeal membrane oxygenation have a role? Pediatrics 2003;112:1274–1278.

10 Williams GD, Numa A, Sokol J, et al. ECLS in pertussis: does it have a role? Intensive Care Med1998;24:1089–1092.

11 Wortis N, Strebel PM, Wharton M, et al. Pertussis deaths: report of 23 cases in the United States, 1992 and 1993. Pediatrics 1996;97:607–612.

12 Sreenan CD, Osiovich H. Neonatal pertussis requiring extracorporeal membrane oxygenation. Pediatr Surg Int 2001;17:201–203.

13 Chan L. Cough and respiratory distress in a two-month-old infant. Pediatr Infect Dis J 1997;16:631–635.

14 Gillis J, Grattan-Smith T, Kilham H. Artificial ventilation in severe pertussis. Arch Dis Child 1988;63:364–367.

15 Goulin GD, Kaya KM, Bradley JS. Severe pulmonary hypertension associated with shock and death in infants infected with Bordetella pertussis. Crit Care Med 1993;21:1791–1794.

16 Pierce C, Klein N, Peters M. Is leukocytosis a predictor of mortality in severe pertussis infection? Intensive Care Med 2000;26:1512–1514.

17 Centers for Disease Control and Prevention. Pertussis deaths: United States 2000. MMWR Morb Mortal Wkly Rep 2002;51:616–618.

18 Johnston ID, Bland JM, Ingram D, et al. Effect of whooping cough in infancy on subsequent lung function and bronchial reactivity. Am Rev Respir Dis 1986;134:270–275.

19 Olsen M, Thygesen SK, Østergaard JR, et al. Hospital-diagnosed pertussis infection in children and long-term risk of epilepsy. JAMA 2015;314:1844-1849.

20 Centers for Disease Control and Prevention. Clinical complications: infants and children. Available at http://www.cdc.gov/pertussis/clinical/complications.html. Accessed April 25, 2020.

21 Zellweger H. Pertussis encephalopathy. Arch Pediatr 1959;76:381–386.

22 Grant CC, McKay EJ, Simpson A, et al. Pertussis encephalopathy with high cerebrospinal fluid antibody titers to pertussis toxin and filamentous hemagglutinin. Pediatrics 1998;102(4 pt 1):986–990.

23 Mulholland EK: Pertussis. In Mabey D, Gill G, Weber MV, et al (eds): Principles of Medicine in Africa. Cambridge, Cambridge University Press, 2013, p 147.

24 World Health Organization. International travel and health: pertussis. Available at http://who.int/ith/diseases/pertussis/en/. Accessed April 25, 2020.

25 Senzilet LD, Halperin SA, Spika JS, et al. Pertussis is a frequent cause of prolonged cough illness in adults and adolescents. Clin Infect Dis 2001; 32:1691–7.

26 Postels-Multani S, Schmitt HJ, Wirsing von König CH, et al. Symptoms and complications of pertussis in adults. Infection 1995;23:139–142.

27 Lee GM, Lett S, Schauer S, et al. Societal costs and morbidity of pertussis in adolescents and adults. Clin Infect Dis 2004;39:1572-80.

28 Eidlitz-Markus T, Zeharia A. Bordetella pertussis as a trigger of migraine without aura. Pediatr Neurol 2005;33:283–284.

29 Skowronski DM, Buxton JA, Hestrin M, et al. Carotid artery dissection as a possible severe complication of pertussis in an adult: clinical case report and review. Clin Infect Dis 2003;36:e1–4.

30 Jenkins P, Clarke SW. Cough syncope: a complication of adult whooping cough. Br J Dis Chest 1981;75:311–313.

31 Halperin SA, Marrie TJ. Pertussis encephalopathy in an adult: case report and review. Rev Infect Dis 1991;13:1043–1047.

32 MacLean DW. Adults with pertussis. J R Coll Gen Pract 1982;32:298–300.

33 Broder KR, Cortese MM, Iskander JK, et al. Preventing tetanus, diphtheria, and pertussis among adolescents: use of tetanus toxoid, reduced diphtheria toxoid and acellular pertussis vaccines. Recommendations of the Advisory Committee on Immunization Practices (ACIP). MMWR Recomm Rep 2006;55(RR-3):1–34.

34 Centers for Disease Control and Prevention. Fatal case of unsuspected pertussis diagnosed from a blood culture: Minnesota, 2003. MMWR Morb Mortal Wkly Rep 2004;53:131–132.

35 Mertens PL, Borsboom GJ, Richardus JH. A pertussis outbreak associated with social isolation among elderly nuns in a convent. Clin Infect Dis 2007;44:266–268.

36 Cherry J, Brunnel P, Golden G. Report of the Task Force on Pertussis and Pertussis Immunization, 1988. Pediatrics 1988;81(Suppl.):933–984.

37 Schmitt-Grohe S, Cherry JD, Heininger U, et al. Pertussis in German adults. Clin Infect Dis 1995;21:860–866.

38 Mink CM, Cherry JD, Christenson P, et al. A search for Bordetella pertussis infection in university students. Clin Infect Dis 1992;14:464–471.

39 Baughman AL, Bisgard KM, Edwards KM, et al. Establishment of diagnostic cutoff points for levels of serum antibodies to pertussis toxin, filamentous hemagglutinin, and

fimbriae in adolescents and adults in the United States. Clin Diagn Lab Immunol 2004;11:1045–1053.

40 Strebel P, Nordin J, Edwards K, et al. Population-based incidence of pertussis among adolescents and adults, Minnesota, 1995–1996. J Infect Dis 2001;183:1353–1359.

41 Cherry JD, Beer T, Chartrand SA, et al. Comparison of values of antibody to Bordetella pertussis antigens in young German and American men. Clin Infect Dis 1995;20:127–1274,.

42 Rosenthal S, Strebel P, Cassiday P, et al. Pertussis infection among adults during the 1993 outbreak in Chicago. J Infect Dis 1995;171:1650–1652.

43 Robertson PW, Goldberg H, Jarvie BH, et al. Bordetella pertussis infection: a cause of persistent cough in adults. Med J Aust 1987;146:522–525.

44 Wright SW, Edwards KM, Decker MD, et al. Pertussis infection in adults with persistent cough. JAMA 1995;273:1044–1046.

45 Nennig ME, Shinefield HR, Edwards KM, et al. Prevalence and incidence of adult pertussis in an urban population. JAMA 1996;275:1672–1674.

46 Centers for Disease Control and Prevention. Pertussis: United States, 1997–2000. MMWR Morb Mortal Wkly Rep 2002;51:73–76.

47 Guris D, Strebel PM, Bardenheier B, et al. Changing epidemiology of pertussis in the United States: increasing reported incidence among adolescents and adults, 1990–1996. Clin Infect Dis 1999;28:1230–1237.

48 National Immunization Program, Bacterial Vaccine Preventable Diseases Branch. Pertussis Surveillance Report, August 6, 2004.

49. World Health Organization. Pertussis global annual reported cases and DTP3 coverage 1980-2018. Available at https://www.who.int/immunization/monitoring_surveillance/burden/vpd/surveillance_type/passive/pertussis_coverage_2018.jpg?ua=1. Accessed July 18, 2020.

50. Chitkara AJ, Pujadas Ferrer M, Forsyth K, et al. Pertussis vaccination in mixed markets: Recommendations from the Global Pertussis Initiative. Int J Infect Dis 2020;96:482-488. doi:10.1016/j.ijid.2020.04.081.

51. Mosser JF, Gagne-Maynard W, Rao PC, et al. Mapping diphtheria-pertussis-tetanus vaccine coverage in Africa, 2000-2016: a spatial and temporal modelling study. Lancet. 2019;393:1843-1855. doi:10.1016/S0140-6736(19)30226-0.

52. Son S, Thamlikitkul V, Chokephaibulkit K, et al. Prospective multinational serosurveillance study of Bordetella pertussis infection among 10- to 18-year-old Asian children and adolescents. Clin Microbiol Infect. 2019;25:250.e1-250.e7. doi:10.1016/j.cmi.2018.04.013.

53. Thisyakorn U, Tantawichien T, Thisyakorn C, Buchy P. Pertussis in the Association of Southeast Asian Nations: epidemiology and challenges. Int J Infect Dis. 2019;87:75-83. doi:10.1016/j.ijid.2019.07.016.

54. Pneumonia Etiology Research for Child Health (PERCH) Study Group. Causes of severe pneumonia requiring hospital admission in children without HIV infection from Africa and Asia: the PERCH multi-country case-control study [published correction appears in Lancet. 2019 Aug 31;394(10200):736]. Lancet. 2019;394:757-779. doi:10.1016/S0140-6736(19)30721-4.

55 Octavia S, Sintchenko V, Gilbert GL, et al. Newly emerging clones of Bordetella pertussis carrying prn2 and ptxP3 alleles implicated in Australian pertussis epidemic in 2008-2010. J Infect Dis 2012;205:1220–1224.

56 Weber C, Boursaux-Eude C, Coralie G, et al. Polymorphism of Bordetella pertussis isolates circulating for the last 10 years in France, where a single effective whole-cell vaccine has been used for more than 30 years. J Clin Microbiol 2001;39:4396–4403.

57 King AJ, Berbers G, van Oirschot HF, et al. Role of the polymorphic region 1 of the Bordetella pertussis protein pertactin in immunity. Microbiology 2001;147:2885–2895.

58 Mooi FR, van Loo IH, King AJ. Adaptation of Bordetella pertussis to vaccination: a cause for its reemergence? Emerg Infect Dis 2001;7(3 Suppl.):526–528.

59 Hallander HO, Advani A, Donnelly D, et al. Shifts of Bordetella pertussis variants in Sweden from 1970 to 2003, during three periods marked by different vaccination programs. J Clin Microbiol 2005;43:2856–2865.

60 Bouchez V, Brun D, Cantinelli T, et al. First report and detailed characterization of B. pertussis isolates not expressing pertussis toxin or pertactin. Vaccine 2009;27:6034–6041.

61 Lam C, Octavia S, Ricafort L, et al. Rapid increase in pertactin-deficient Bordetella pertussis isolates, Australia. Emerg Infect Dis 2014;20:626-633.

62 Hegerle N, Dore G, Guiso N. Pertactin deficient Bordetella pertussis present a better fitness in mice immunized with an acellular pertussis vaccine. Vaccine 2014;32:6597-6600.

63 Zeddeman A, van Gent M, Heuvelman CJ, et al. Investigations into the emergence of pertactin-deficient Bordetella pertussis isolates in six European countries, 1996 to 2012. Euro Surveill 2014;19:20881.

64 Pawloski LC, Queenan AM, Cassiday PK, et al. Prevalence and molecular characterization of pertactin-deficient Bordetella pertussis in the United States. Clin Vaccine Immunol 2014;21:119-125.

65 Martin SW, Pawloski L, Williams M, et al. Pertactin-negative Bordetella pertussis strains: evidence for a possible selective advantage. Clin Infect Dis 2015;60:223-227.

66 Weigand MR, Williams MM, Peng Y, Kania D, Pawloski LC, Tondella ML; CDC Pertussis Working Group. Genomic Survey of Bordetella pertussis Diversity, United States, 2000-2013. Emerg Infect Dis 2019;25:780-783. doi: 10.3201/eid2504.180812.

67 Breakwell L, Kelso P, Finley C, et al. Pertussis vaccine effectiveness in the setting of pertactin-deficient pertussis. Pediatrics 2016;137:e20153973.

68 Tuomanen E: Bordetella pertussis adhesins. In: Wardlaw AC, Parton R (eds): Pathogenesis and Immunity in Pertussis. New York, John Wiley & Sons, 1988, pp 75–94.

69 Tuomanen E, Weiss A, Rich R, et al. Filamentous hemagglutinin and pertussis toxin promote adherence of Bordetella pertussis to cilia. Dev Biol Stand 1985;61:197–204.

70 Brennan MJ, Li ZM, Cowell JL, et al. Identification of a 69-kilodalton nonfimbrial protein as an agglutinogen of Bordetella pertussis. Infect Immun 1988;56:3189–3195.

71 Hewlett EL. Pertussis: current concepts of pathogenesis and prevention. Pediatr Infect Dis J 1997;16(4 Suppl.):S78–84.

72 Weiss A. Mucosal immune defenses and the response of Bordetella pertussis. ASM News 1997;63:22–28.

73 Zhang JM, Cowell JL, Steven AC, et al. Purification of serotype 2 fimbriae of Bordetella pertussis and their identification as a mouse protective antigen. Dev Biol Stand 1985;61:173–185.

74 de Gouw D, Diavatopoulos DA, Bootsma HJ, et al. Pertussis: a matter of immune modulation. FEMS Microbiol Rev 2011;35:441–474.

75 Melvin JA, Scheller EV, Miller JF, et al. Bordetella pertussis pathogenesis: current and future challenges. Nat Rev Microbiol 2014;12:274-288.

76 Warfel JM, Beren J, Kelly VK, et al. Nonhuman primate model of pertussis. Infect Immun 2012;80:1530-1536.

77 Warfel JM, Beren J, Merkel TJ. Airborne transmission of Bordetella pertussis. J Infect Dis 2012;206:902-906.

78 Warfel JM, Zimmerman LI, Merkel TJ. Acellular pertussis vaccines protect against disease but fail to prevent infection and transmission in a nonhuman primate model. Proc Natl Acad Sci USA 2014;111:787-792.

79 Long SS, Welkon CJ, Clark JL. Widespread silent transmission of pertussis in families: antibody correlates of infection and symptomatology. J Infect Dis 1990;161:480–486.

80 Waters V, Jamieson F, Richardson SE, et al. Outbreak of atypical pertussis detected by polymerase chain reaction in immunized preschool-aged children. Pediatr Infect Dis J 2009;28:582–587.

81 Klement E, Uliel L, Engel I, et al. An outbreak of pertussis among young Israeli soldiers. Epidemiol Infect 2003;131:1049–1054.

82 Zhang Q, Yin Z, Li Y, Luo H, et al. Prevalence of asymptomatic Bordetella pertussis and Bordetella parapertussis infections among school children in China as determined by pooled real-time PCR: a cross-sectional study. Scand J Infect Dis 2014;46:280-287.

83 Srugo I, Benilevi D, Madeb R, et al. Pertussis infection in fully vaccinated children in day-care centers, Israel. Emerg Infect Dis 2000;6:526–529.

84 Fisher MC, Long SS, McGowan KL, et al. Outbreak of pertussis in a residential facility for handicapped people. J Pediatr 1989;114:934–939.

85 Craig R, Kunkel E, Crowcroft NS, et al. Asymptomatic Infection and Transmission of Pertussis in Households: A Systematic Review. Clin Infect Dis 2020;70:152-161. doi: 10.1093/cid/ciz531.

86 Broome CV, Fraser DW, English WJ: Pertussis: diagnostic methods and surveillance. In Manclark CR, Hill JC (eds): International Symposium on Pertussis. Bethesda, MD, US Department of Health, Education, and Welfare, Public Health Service, National Institutes of Health, DHEW publication no. (NIH)79-1830:19–22. 1979.

87 Strebel PM, Cochi SL, Farizo KM, et al. Pertussis in Missouri: evaluation of nasopharyngeal culture, direct fluorescent antibody testing, and clinical case definitions in the diagnosis of pertussis. Clin Infect Dis 1993;16:276–285.

88 Onorato IM, Wassilak SG. Laboratory diagnosis of pertussis: the state of the art. Pediatr Infect Dis J 1987;6:145–151.

89 Halperin SA, Bortolussi R, Wort AJ. Evaluation of culture, immunofluorescence, and serology for the diagnosis of pertussis. J Clin Microbiol 1989;27:752–757.

90 Hallander HO, Reizenstein E, Renemar B, et al. Comparison of nasopharyngeal aspirates with swabs for culture of Bordetella pertussis. J Clin Microbiol 1993;31:50–52.

91 Cattaneo LA, Reed GW, Haase DH, et al. The seroepidemiology of Bordetella pertussis infections: a study of persons ages 1–65 years. J Infect Dis 1996;173:1256–1259.

92 Jõgi P, Oona M, Toompere K, et al. Seroprevalence of IgG antibodies to pertussis toxin in children and adolescents in Estonia. Vaccine 2014;32:5311-5315.

93 Zhang Q, Zheng H, Liu M, et al. The seroepidemiology of immunoglobulin G antibodies against pertussis toxin in China: a cross sectional study. BMC Infect Dis 2012;12:138.

94 Rendi-Wagner P, Tobias J, Moerman L, et al. The seroepidemiology of Bordetella pertussis in Israel--Estimate of incidence of infection. Vaccine 2010;28:3285-3290.

95 Wang CQ, Zhu QR. Seroprevalence of Bordetella pertussis antibody in children and adolescents in China. Pediatr Infect Dis J 2011;30:593-596.

96 Mossong J, Putz L, Shkedy Z, et al. Seroepidemiology of diphtheria and pertussis in Luxembourg in 2000. Epidemiol Infect 2006;134:573-578.

97 Giammanco A, Chiarini A, Stroffolini T, et al. Seroepidemiology of pertussis in Italy. Rev Infect Dis 1991;13:1216–1220.

98 Cherry JD, Chang SJ, Klein D, et al. Prevalence of antibody to Bordetella pertussis antigens in serum specimens obtained from 1793 adolescents and adults. Clin Infect Dis 2004;39:1715–1718.

99 de Greeff SC, de Melker HE, van Gageldonk PG, et al. Seroprevalence of pertussis in the Netherlands: evidence for increased circulation of Bordetella pertussis. PLoS One 2010;5:e14183.

100 Halperin SA, Bortolussi R, MacLean D, et al. Persistence of pertussis in an immunized population: results of the Nova Scotia Enhanced Pertussis Surveillance Program. J Pediatr 1989;115:686–693.

101 Steketee RW, Burstyn DG, Wassilak SG, et al. A comparison of laboratory and clinical methods for diagnosing pertussis in an outbreak in a facility for the developmentally disabled. J Infect Dis 1988;157:441–449.

102 Addiss DG, Davis JP, Meade BD, et al. A pertussis outbreak in a Wisconsin nursing home. J Infect Dis 1991;164:704–710.

103 Marchant CD, Loughlin AM, Lett SM, et al. Pertussis in Massachusetts, 1981–1991: incidence, serologic diagnosis, and vaccine effectiveness. J Infect Dis 1994;169:1297–1305.

104 Centers for Disease Control and Prevention. Pertussis (whooping cough) diagnostic testing. Available at https://www.cdc.gov/pertussis/clinical/diagnostic-testing/index.html. Accessed April 25, 2020.

105 Yih WK, Lett SM, des Vignes FN, et al. The increasing incidence of pertussis in Massachusetts adolescents and adults, 1989–1998. J Infect Dis 2000;182:1409–1416.

106 de Melker HE, Versteegh FG, Conyn-Van Spaendonck MA, et al. Specificity and sensitivity of high levels of immunoglobulin G antibodies against pertussis toxin in a single serum sample for diagnosis of infection with Bordetella pertussis. J Clin Microbiol 2000;38:800–806.

107 Wirsing von König CH, Schmitt HJ, Bogaerts H, et al. Factors influencing the analysis of secondary prevention of pertussis. Dev Biol Stand 1997;89:175–179.

108 De Serres G, Boulianne N, Duval B. Field effectiveness of erythromycin prophylaxis to prevent pertussis within families. Pediatr Infect Dis J 1995;14:969–975.

109 Winter K, Zipprich J, Harriman K, et al. Risk factors associated with infant deaths from pertussis: a case-control study. Clin Infect Dis 2015;61:1099-1106.

110 Tiwari T, Murphy TV, Moran J. Recommended antimicrobial agents for the treatment and postexposure prophylaxis of pertussis: 2005 CDC Guidelines. MMWR Recomm Rep 2005;54(RR-14):1–16.

111 Kimberlin DW, Brady MT, Jackson MA, et al, eds. Red book: 2015 report of the Committee on Infectious Diseases. 30th ed. Elk Grove Village, IL: American Academy of Pediatrics, 2015.

112 Halperin SA, Bortolussi R, Langley JM, et al. Seven days of erythromycin estolate is as effective as fourteen days for the treatment of Bordetella pertussis infections. Pediatrics 1997;100:65–71.

113 Bass JW, Klenk EL, Kotheimer JB, et al. Antimicrobial treatment of pertussis. J Pediatr 1969;75:768–781.

114 Hewlett EL: Bordetella species. In Mandell G (ed): Douglas and Bennett's Principles and Practice of Infectious Diseases. 4th ed. New York, Churchill Livingstone, 1995.

115 Mortimer Jr EA, Jones PK. An evaluation of pertussis vaccine. Rev Infect Dis 1979;1:927–934.

116 Kawai H, Aoyama T, Goto A, et al. Evaluation of pertussis treatment with erythromycin ethylsuccinate and stearate according to age. Kansenshogaku Zasshi 1994;68:1324–1329.

117 Aoyama T, Sunakawa K, Iwata S, et al. Efficacy of short-term treatment of pertussis with clarithromycin and azithromycin. J Pediatr 1996;129:761–764.

118 Halperin SA, Bortolussi R, Langley JM, et al. A randomized, placebo-controlled trial of erythromycin estolate chemoprophylaxis for household contacts of children with culture-positive Bordetella pertussis infection. Pediatrics 1999;104:e42.

119 Bortolussi R, Miller B, Ledwith M, et al. Clinical course of pertussis in immunized children. Pediatr Infect Dis J 1995;14:870–874.

120 Li L, Deng J, Ma X, et al. High Prevalence of Macrolide-Resistant Bordetella pertussis and ptxP1 Genotype, Mainland China, 2014-2016. Emerg Infect Dis 2019; 25:2205-14.

121 Kwantes W, Joynson DH, Williams WO. Bordetella pertussis isolation in general practice: 1977–79 whooping cough epidemic in West Glamorgan. J Hyg (Lond) 1983;90:149–158.

122 Henry R, Dorman D, Skinner J, et al. Limitations of erythromycin in whooping cough. Med J Aust 1981;2:108–109.

123 Beneson AS. Control of Communicable Disease Manual. 16th ed. Washington, DC: American Public Health Association; 1995.

124 Madsen T. Vaccination against whooping cough. JAMA 1933;101:187–188.

125 Kendrick P, Eldering G. Progress report on pertussis immunization. Am J Public Health 1936;26:8–12.

126 Schmitt HJ, von Konig CH, Neiss A, et al. Efficacy of acellular pertussis vaccine in early childhood after household exposure. JAMA 1996;275:37–41.

127 Liese JG, Meschievitz CK, Harzer E, et al. Efficacy of a two-component acellular pertussis vaccine in infants. Pediatr Infect Dis J 1997;16:1038–1044.

128 Ramsay ME, Farrington CP, Miller E. Age-specific efficacy of pertussis vaccine during epidemic and non-epidemic periods. Epidemiol Infect 1993;111:41–48.

129 Gustafsson L, Hallander HO, Olin P, et al. A controlled trial of a two-component acellular, a five-component acellular, and a whole-cell pertussis vaccine. N Engl J Med 1996;334:349–355.

130 Greco D, Salmaso S, Mastrantonio P, et al. A controlled trial of two acellular vaccines and one whole-cell vaccine against pertussis. Progetto Pertosse Working Group. N Engl J Med 1996;334:341–348.

131 Simondon F, Preziosi MP, Yam A, et al. A randomized double-blind trial comparing a two-component acellular to a whole-cell pertussis vaccine in Senegal. Vaccine 1997;15:1606–1612.

132 Kanai K. Japan's experience in pertussis epidemiology and vaccination in the past thirty years. Jpn J Med Sci Biol 1980;33:107–143.

133 Kimura M, Kuno-Sakai H. Immunization system in Japan: its history and present situation. Acta Paediatr Jpn 1988;30:109–126.

134 Sato Y, Kimura M, Fukumi H. Development of a pertussis component vaccine in Japan. Lancet 1984;1:122–126.

135 Cherry JD. The epidemiology of pertussis and pertussis immunization in the United Kingdom and the United States: a comparative study. Curr Probl Pediatr 1984;14:1–78.

136 Stuart-Harris CH: Experiences of pertussis in the United Kingdom. In Manclark CR, Hill JC (eds): International Symposium on Pertussis. Washington, DC, US Department of Health, Education, and Welfare, publication (NIH) 79-1830, 1979.

137 Romanus V, Jonsell R, Bergquist SO. Pertussis in Sweden after the cessation of general immunization in 1979. Pediatr Infect Dis J 1987;6:364–671.

138 Storsaeter J, Olin P. Relative efficacy of two acellular pertussis vaccines during three years of passive surveillance. Vaccine 1992;10:142–144.

139 Taranger J, Trollfors B, Bergfors E, et al. Immunologic and epidemiologic experience of vaccination with a monocomponent pertussis toxoid vaccine. Pediatrics 2001;108:E115.

140 Onorato IM, Wassilak SG, Meade B. Efficacy of whole-cell pertussis vaccine in preschool children in the United States. JAMA 1992;267:2745–2749.

141 Vesselinova-Jenkins CK, Newcombe RG, Gray OP, et al. The effects of immunisation upon the natural history of pertussis: a family study in the Cardiff area. J Epidemiol Community Health 1978;32:194–199.

142 Grob PR, Crowder MJ, Robbins JF. Effect of vaccination on severity and dissemination of whooping cough. Br Med J (Clin Res Ed) 1981;282:1925–1928.

143 Preziosi MP, Halloran ME. Effects of pertussis vaccination on disease: vaccine efficacy in reducing clinical severity. Clin Infect Dis 2003;37:772–779.

144 Preziosi MP, Halloran ME. Effects of pertussis vaccination on transmission: vaccine efficacy for infectiousness. Vaccine 2003;21:1853–1861.

145 McNamara LA, Skoff T, Faulkner A, et al. Reduced Severity of Pertussis in Persons With Age-Appropriate Pertussis Vaccination-United States, 2010-2012. Clin Infect Dis 2017;65:811-818. doi: 10.1093/cid/cix421.

146 Centers for Disease Control and Prevention. Diphtheria, tetanus, and pertussis: recommendations for vaccine use and other preventive measures. Recommendations of the Immunization Practices Advisory Committee (ACIP). MMWR Recomm Rep 1991;40(RR-10):1–28.

147 Prymula R, Siegrist CA, Chlibek R, et al. Effect of prophylactic paracetamol administration at time of vaccination on febrile reactions and antibody responses in children: two open-label, randomised controlled trials. Lancet 2009;374:1339-1350.

148 Cadoz M, Arminjon F, Quentin-Millet MJ: Safety and immunogenicity of Merieux acellular pertussis in adult volunteers. In Transcript of the Workshop on Acellular

Pertussis Vaccines. Washington, DC, US Department of Health and Human Services, 1986, pp 131–134.

149 Cody CL, Baraff LJ, Cherry JD, et al. Nature and rates of adverse reactions associated with DTP and DT immunizations in infants and children. Pediatrics 1981;68:650–660.

150 Decker MD, Edwards KM, Steinhoff MC, et al. Comparison of 13 acellular pertussis vaccines: adverse reactions. Pediatrics 1995;96:557–566.

151 Baraff LJ, Cody CL, Cherry JD. DTP-associated reactions: an analysis by injection site, manufacturer, prior reactions, and dose. Pediatrics 1984;73:31–36.

152 Berkovic SF, Harkin L, McMahon JM, et al. De-novo mutations of the sodium channel gene SCN1A in alleged vaccine encephalopathy: a retrospective study. Lancet Neurol 2006;5:488-492.

153 Reyes IS, Hsieh DT, Laux LC, et al. Alleged cases of vaccine encephalopathy rediagnosed years later as Dravet syndrome. Pediatrics 2011;128:e699-702.

154 Howson CP, Fineberg HV. Adverse events following pertussis and rubella vaccines: summary of a report of the Institute of Medicine. JAMA 1992;267:392–396.

155 DPT Vaccine and Chronic Nervous System Dysfunction: A New Analysis. Institute of Medicine (US) Committee to Study New Research on Vaccines; Stratton KR, Howe CJ, Johnston RB Jr., editors. Washington (DC): National Academies Press (US); 1994.

156 Geraghty K. Presentation to the Immunization Practices Advisory Committee, Centers for Disease Control and Prevention. Atlanta, GA, October 25, 1985.

157 Howson CP, Fineberg HV. The ricochet of magic bullets: summary of the Institute of Medicine Report, Adverse Effects of Pertussis and Rubella Vaccines. Pediatrics 1992;89:318–324.

158 Kimura M, Hikino N. Results with a new DTP vaccine in Japan. Dev Biol Stand 1985;61:545–561.

159 Aoyama T, Murase Y, Gonda T, et al. Type-specific efficacy of acellular pertussis vaccine. Am J Dis Child 1988;142:40–42.

160 Kimura M, Kuno-Sakai H. Current epidemiology of pertussis in Japan. Pediatr Infect Dis J 1990;9:705–709.

161 Noble GR, Bernier RH, Esber EC, et al. Acellular and whole-cell pertussis vaccines in Japan: report of a visit by US scientists. JAMA 1987;257:1351–1356.

162 Kimura M. Japanese clinical experiences with acellular pertussis vaccines. Dev Biol Stand 1991;73:5–9.

163 Kuno-Sakai H, Kimura M. Safety and efficacy of acellular pertussis vaccine in Japan, evaluated by 23 years of its use for routine immunization. Pediatr Int 2004;46:650–655.

164 Okada K, Ohashi Y, Matsuo F, et al. Effectiveness of an acellular pertussis vaccine in Japanese children during a non-epidemic period: a matched case-control study. Epidemiol Infect 2009;137:124-130.

165 Ohfuji S, Okada K, Nakano T, et al. Effectiveness of acellular pertussis vaccine in a routine immunization program: a multicenter, case-control study in Japan. Vaccine 2015;33:1027-1032.

166 Klein DL. Multicenter acellular pertussis vaccine trial: a National Institutes of Health perspective. Pediatrics 1995;96 (Pt 2):547-8.

167 Edwards KM, Meade BD, Decker MD, et al. Comparison of 13 acellular pertussis vaccines: overview and serologic response. Pediatrics 1995;96:548–557.

168 Edwards KM, Decker MD. Pertussis Vaccine. In: Plotkin SA, Orenstein WA, Offit PA, Edwards KM, eds. Vaccines, 7th ed. Philadelphia: Elsevier, 2017:711-761.

169 Placebo-controlled trial of two acellular pertussis vaccines in Sweden: protective efficacy and adverse events. Ad Hoc Group for the Study of Pertussis Vaccines. Lancet 1988;1:955–960.

170 Storsaeter J, Olin P, Renemar B, et al. Mortality and morbidity from invasive bacterial infections during a clinical trial of acellular pertussis vaccines in Sweden. Pediatr Infect Dis J 1988;7:637–645.

171 Blackwelder WC, Storsaeter J, Olin P, et al. Acellular pertussis vaccines. Efficacy and evaluation of clinical case definitions. Am J Dis Child 1991;145:1285–1289.

172 Olin P, Storsaeter J. Vaccine efficacy in a clinical trial of acellular pertussis vaccines in Sweden: technical report. Stockholm, Sweden, National Bacteriological Laboratory, 1988.

173 Storsaeter J, Hallander H, Farrington CP, et al. Secondary analyses of the efficacy of two acellular pertussis vaccines evaluated in a Swedish phase III trial. Vaccine 1990;8:457–461.

174 Trollfors B, Taranger J, Lagergard T, et al. A placebo-controlled trial of a pertussis-toxoid vaccine. N Engl J Med 1995;333:1045–1050.

175 Ciofi degli Atti ML, Olin P. Severe adverse events in the Italian and Stockholm I pertussis vaccine clinical trials. Dev Biol Stand 1997;89:77–81.

176 Sanofi Pasteur. Tripedia® package insert. Available at https://www.vaccineshoppe.com/assets/pdf/tripedia.pdf. Accessed April 25, 2020.

177 Stehr K, Cherry JD, Heininger U, et al. A comparative efficacy trial in Germany in infants who received either the Lederle/Takeda acellular pertussis component DTP (DTaP) vaccine, the Lederle whole-cell component DTP vaccine, or DT vaccine. Pediatrics 1998;101:1–11.

178 Taranger J, Trollfors B, Bergfors E, et al. Mass vaccination of children with pertussis toxoid–decreased incidence in both vaccinated and nonvaccinated persons. Clin Infect Dis 2001;33:1004–1010.

179 Trollfors B, Dotevall L, Sundh V, et al. Pertussis after end of a mass vaccination project: end of the “vaccination honey-moon”. Vaccine 2011;29:2444–2450.

180 Public Health Agency of Sweden. Pertussis surveillance in Sweden: twenty year report. Available at https://www.folkhalsomyndigheten.se/contentassets/14d7068f02604bb496a319895fd9ad47/pertussis-sweden-twenty-year-report-18068.pdf. Accessed April 25, 2020.

181 Gustafsson L, Hallander H, Olin P. Pertussis surveillance in Sweden: progress report Oct 1997-Sept 2004 with executive summary: seven year report. Stockholm, Swedish Institute for Infectious Disease Control (SMI), 2005.

182 Gustafsson L, Hessel L, Storsaeter J, et al. Long-term follow-up of Swedish children vaccinated with acellular pertussis vaccines at 3, 5, and 12 months of age indicates the need for a booster dose at 5 to 7 years of age. Pediatrics 2006;118:978–984.

183 Thierry-Carstensen B, Dalby T, Stevner MA, Robbins JB, Schneerson R, Trollfors B. Experience with monocomponent acellular pertussis combination vaccines for infants, children, adolescents and adults--a review of safety, immunogenicity, efficacy and effectiveness studies and 15 years of field experience. Vaccine 2013; 31:5178-91.

184 Tiwari TS, Baughman AL, Clark TA. First pertussis vaccine dose and prevention of infant mortality. Pediatrics. 2015 Jun;135(6):990-9. doi: 10.1542/peds.2014-2291. Epub 2015 May 4.

185 Olin P, Gustafsson L, Rasmussen F. Efficacy trial of acellular pertussis vaccines: technical report trial II, with results of preplanned analysis of efficacy and immunogenicity and safety. Stockholm, Sweden, Swedish Institute for Infectious Disease Control, 1997.

186 Blumberg DA, Mink CM, Cherry JD, et al. Comparison of acellular and whole-cell pertussis-component diphtheria-tetanus-pertussis vaccines in infants. The APDT Vaccine Study Group. J Pediatr 1991;119:194–204.

187 Edwards KM, Karzon DT. Pertussis vaccines. Pediatr Clin North Am 37:549–566, 1990.

188 Lewis K, Cherry JD, Holroyd HJ, et al. A double-blind study comparing an acellular pertussis-component DTP vaccine with a whole-cell pertussis-component DTP vaccine in 18-month-old children. Am J Dis Child 1986;140:872–876.

189 Edwards KM, Lawrence E, Wright PF. Diphtheria, tetanus, and pertussis vaccine: a comparison of the immune response and adverse reactions to conventional and acellular pertussis components. Am J Dis Child 1986;140:867–871.

190 Edwards KM, Bradley RB, Decker MD, et al. Evaluation of a new highly purified pertussis vaccine in infants and children. J Infect Dis 1989;160:832–837.

191 Pichichero ME, Badgett JT, Rodgers Jr GC, et al. Acellular pertussis vaccine: immunogenicity and safety of an acellular pertussis vs. a whole cell pertussis vaccine

combined with diphtheria and tetanus toxoids as a booster in 18- to 24-month-old children. Pediatr Infect Dis J 1987;6:352–363.

192 Anderson EL, Belshe RB, Bartram J. Differences in reactogenicity and antigenicity of acellular and standard pertussis vaccines combined with diphtheria and tetanus in infants. J Infect Dis 1988;157:731–737.

193 Moro PL, Perez-Vilar S, Lewis P, Bryant-Genevier M, Kamiya H, Cano M. Safety Surveillance of Diphtheria and Tetanus Toxoids and Acellular Pertussis (DTaP) Vaccines. Pediatrics 2018;142. pii: e20174171. doi: 10.1542/peds.2017-4171. Epub 2018 Jun 4.

194 Pichichero ME, Deloria MA, Rennels MB, et al. A safety and immunogenicity comparison of 12 acellular pertussis vaccines and one whole-cell pertussis vaccine given as a fourth dose in 15- to 20-month-old children. Pediatrics 1997;100:772–788.

195 Pichichero ME, Edwards KM, Anderson EL, et al. Safety and immunogenicity of six acellular pertussis vaccines and one whole-cell pertussis vaccine given as a fifth dose in four- to six-year-old children. Pediatrics 2000;105:e11.

196 Rennels MB, Deloria MA, Pichichero ME, et al. Extensive swelling after booster doses of acellular pertussis-tetanus-diphtheria vaccines. Pediatrics 2000;105:e12.

197 Schmitt HJ, Beutel K, Schuind A, et al. Reactogenicity and immunogenicity of a booster dose of a combined diphtheria, tetanus, and tricomponent acellular pertussis vaccine at fourteen to twenty-eight months of age. J Pediatr 1997;130:616–623.

198 Liese JG, Stojanov S, Zink TH, et al. Safety and immunogenicity of Biken acellular pertussis vaccine in combination with diphtheria and tetanus toxoid as a fifth dose at four to six years of age. Munich Vaccine Study Group. Pediatr Infect Dis J 2001;20:981–988.

199 Rennels MB, Deloria MA, Pichichero ME, et al. Lack of consistent relationship between quantity of aluminum in diphtheria-tetanus-acellular pertussis vaccines and rates of extensive swelling reactions. Vaccine 2002;20(Suppl. 3):S44–47.

200 Isomura S. Efficacy and safety of acellular pertussis vaccine in Aichi Prefecture, Japan. Pediatr Infect Dis J 1988;7:258–262.

201 Blennow M, Granstrom M. Adverse reactions and serologic response to a booster dose of acellular pertussis vaccine in children immunized with acellular or whole-cell vaccine as infants. Pediatrics 1989;84:62–67.

202 Scheifele DW, Halperin SA, Ochnio JJ, et al. A modified vaccine reduces the rate of large injection site reactions to the preschool booster dose of diphtheria-tetanus-acellular pertussis vaccine: results of a randomized, controlled trial. Pediatr Infect Dis J 2005;24:1059–1066.

203 Knuf M, Habermehl P, Faber J, et al. Assessment of nine candidate DTP-vaccines with reduced amount of antigen and/or without adjuvant as a fourth (booster-) dose in the second year of life. Vaccine 2006;24:5627–5636.

204 Rennels MB, Black S, Woo EJ, et al. Safety of a fifth dose of diphtheria and tetanus toxoid and acellular pertussis vaccine in children experiencing extensive, local reactions to the fourth dose. Pediatr Infect Dis J 2008;27:464-465.

206 Keitel WA, Muenz LR, Decker MD, et al. A randomized clinical trial of acellular pertussis vaccines in healthy adults: dose-response comparisons of 5 vaccines and implications for booster immunization. J Infect Dis 1999;180:397–403.

207 Decker MD, Cates KL, Maida A. Safety and immunogenicity in adults and adolescents of Biocine acellular pertussis (aP) vaccine. In Abstracts of the 7th International Congress for Infectious Diseases, Hong Kong, June, 1996, Abstract 123.002.

208 Edwards KM, Decker MD, Graham BS, et al. Adult immunization with acellular pertussis vaccine. JAMA 1993;269:53–56.

209 Sekura RD, Zhang YL, Roberson R, et al. Clinical, metabolic, and antibody responses of adult volunteers to an investigational vaccine composed of pertussis toxin inactivated by hydrogen peroxide. J Pediatr 1988;113:806–813.

210 Stehr K, Lugauer S, Heninger U. Immunogenicity and reactogenicity of Lederle/Takeda acellular pertussis vaccine in 185 German adults. Pediatr Res 1995;37:189A.

211 Keitel W. Adult pertussis study results using five acellular vaccines. Acellular pertussis vaccine trials: results and impact on US public health. Presented at the National Institutes of Health Pertussis Conference. Washington, DC, June 3–5, 1996.

212 Ward JI, Cherry JD, Chang SJ, et al. Efficacy of an acellular pertussis vaccine among adolescents and adults. N Engl J Med 2005;353:1555–1563.

213 Rank C, Quinn HE, McIntyre PB. pertussis vaccine effectiveness after mass immunization of high school students in Australia. Pediatr Infect Dis 2009;J 28:152-153.

214 Skoff TH, Cohn AC, Clark TA, et al. Early Impact of the US Tdap vaccination program on pertussis trends. Arch Pediatr Adolesc Med 2012;166:344-349.

215 Winter K, Harriman K, Zipprich J, et al. California pertussis epidemic, 2010. J Pediatr 2012;161:1091-1096.

216 Winter K, Glaser C, Watt J, et al. Pertussis epidemic--California, 2014. MMWR Morb Mortal Wkly Rep 2014;63:1129-1132

217 Acosta AM, DeBolt C, Tasslimi A, et al. Tdap vaccine effectiveness in adolescents during the 2012 Washington State pertussis epidemic. Pediatrics 135:981-989, 2015.

218 Liko J, Robison SG, Cieslak PR. Pertussis vaccine performance in an epidemic year-Oregon, 2012. Clin Infect Dis 2014;59:261-263.

219 Liang JL, Cocooning and Tdap vaccination, Advisory Committee on Immunization Practices, Atlanta, GA, June 25, 2015. Data source: CDC, National Notifiable Diseases Surveillance System and Supplemental Pertussis Surveillance System. To obtain slides, make request to acip@cdc.gov.

220 Quinn HE, Snelling TL, Habig A, et al. Parental Tdap boosters and infant pertussis: a case-control study. Pediatrics 2014;134:713-720.

221 Liko J, Robison SG, Cieslak PR. Priming with whole-cell versus acellular pertussis vaccine. N Engl J Med 2013;368:581-582.

222 Carcione D, Regan AK, Tracey L, et al. The impact of parental postpartum pertussis vaccination on infection in infants: A population-based study of cocooning in Western Australia. Vaccine 2015;33:5654-5661.

223 Urwyler P, Heininger U. Protecting newborns from pertussis - the challenge of complete cocooning. BMC Infect Dis 2014;14:397.

224 Healy CM, Rench MA, Wootton SH, et al, Evaluation of the impact of a pertussis cocooning program on infant pertussis infection. Pediatr Infect Dis J 2015;34:22-26.

225 Munoz FM, Bond NH, Maccato M, et al. Safety and immunogenicity of tetanus diphtheria and acellular pertussis (Tdap) immunization during pregnancy in mothers and infants: a randomized clinical trial. JAMA 2014;311:1760-1769.

226 Liang J, Pertussis Working Group. Overview of Tdap use during pregnancy: unpublished and recent data. Presented at the Advisory Committee on Immunization Practices, Atlanta, GA, February 23, 2011.

227 Amirthalingam G, Andrews N, Campbell H, et al. Effectiveness of maternal pertussis vaccination in England: an observational study. Lancet 2014;384:1521-1528.

228 Dabrera G, Amirthalingam G, Andrews N, et al. A case-control study to estimate the effectiveness of maternal pertussis vaccination in protecting newborn infants in England and Wales, 2012-2013. Clin Infect Dis 2015;60:333-337.

229 Halperin SA, Langley JM, Ye L, et al. A randomized controlled trial of the safety and immunogenicity of tetanus, diphtheria, and acellular pertussis vaccine immunization during pregnancy and subsequent infant immune response. Clin Infect Dis 2018;67:1063-1071. doi: 10.1093/cid/ciy244.

230 Skoff TH, Blain AE, Watt J, et al. Impact of the US maternal tetanus, diphtheria, and acellular pertussis vaccination program on preventing pertussis in infants <2 months of age: a case-control evaluation. Clin Infect Dis 2017;65:1977-1983. doi: 10.1093/cid/cix724.

231 Griffin JB, Yu L, Watson D, et al. Pertussis Immunisation in Pregnancy Safety (PIPS) Study: A retrospective cohort study of safety outcomes in pregnant women vaccinated with Tdap vaccine. Vaccine 2018;36:5173-5179. doi: 10.1016/j.vaccine.2018.07.011.

232 Fortner KB, Swamy GK, Broder KR, et al. Reactogenicity and immunogenicity of tetanus toxoid, reduced diphtheria toxoid, and acellular pertussis vaccine (Tdap) in pregnant and nonpregnant women. Vaccine 2018;36:6354-6360. doi: 10.1016/j.vaccine.2018.07.012.

233 Gentile A, Juarez MDV, Lucion MF, et al. Bordetella pertussis (Bp) disease: Before (2003-2011) and after (2013-2016) maternal immunization strategy in a pediatric hospital. Vaccine 2018;36:1375-1380. doi: 10.1016/j.vaccine.2018.01.091.

234 Fernandes EG, Sato APS, Vaz-de-Lima LRA, et al. The effectiveness of maternal pertussis vaccination in protecting newborn infants in Brazil: A case-control study. Vaccine 2019;37:5481-5484. doi: 10.1016/j.vaccine.2019.03.049.

235 Hall C, Abramovitz LM, Bukowinski AT, et al. Safety of tetanus, diphtheria, and acellular pertussis vaccination among pregnant active duty U.S. military women. Vaccine 2020;38:1982-1988. doi: 10.1016/j.vaccine.2020.01.009.

236 Kerr SM, Van Bennekom CM, Mitchell AA. Tetanus, diphtheria, and pertussis vaccine (Tdap) in pregnancy and risk of major birth defects in the offspring. Birth Defects Res 2020;112:393-403. doi: 10.1002/bdr2.1642.

237 Friedrich F, Valadão MC, Brum M, et al. Impact of maternal dTpa vaccination on the incidence of pertussis in young infants. PLoS One 2020;15(1):e0228022. doi: 10.1371/journal.pone.0228022.

238 Langsam D, Anis E, Haas EJ, et al. Tdap vaccination during pregnancy interrupts a twenty-year increase in the incidence of pertussis. Vaccine 2020;38:2700-2706. doi: 10.1016/j.vaccine.2020.01.095.

239 McHugh L, Marshall HS, Perrett KP, et al. The safety of influenza and pertussis vaccination in pregnancy in a cohort of Australian mother-infant pairs, 2012-2015: The FluMum study. Clin Infect Dis 2019;68:402-408. doi: 10.1093/cid/ciy517.

240 Vygen-Bonnet S, Hellenbrand W, Garbe E, vet al. Safety and effectiveness of acellular pertussis vaccination during pregnancy: a systematic review. BMC Infect Dis 2020;20:136. doi: 10.1186/s12879-020-4824-3.

241 Perrett KP, Halperin SA, Nolan T, et al. Immunogenicity, transplacental transfer of pertussis antibodies and safety following pertussis immunization during pregnancy: Evidence from a randomized, placebo-controlled trial. Vaccine 2020;38:2095-2104. doi: 10.1016/j.vaccine.2019.10.105.

242 Romanin V, Acosta AM, Juarez MDV, et al. Maternal Vaccination in Argentina: Tetanus, Diphtheria, and Acellular Pertussis Vaccine Effectiveness During Pregnancy in Preventing Pertussis in Infants <2 Months of Age. Clin Infect Dis 2020;70:380-387. doi: 10.1093/cid/ciz217.

243 Rice TF, Diavatopoulos DA, Smits GP, et al. Antibody responses to Bordetella pertussis and other childhood vaccines in infants born to mothers who received pertussis vaccine in pregnancy - a prospective, observational cohort study from the United Kingdom. Clin Exp Immunol 2019;197:1-10. doi: 10.1111/cei.13275.

244 Zimmermann P, Perrett KP, Messina NL, et al. The Effect of Maternal Immunisation During Pregnancy on Infant Vaccine Responses. EClinicalMedicine 2019;13:21-30. doi: 10.1016/j.eclinm.2019.06.010.

245 World Health Organization. WHO vaccine-preventable diseases: monitoring system. 2019 global summary. Available at https://apps.who.int/immunization_monitoring/globalsummary/schedules. Accessed April 25, 2020.

246 Mazzilli S, Tavoschi L, Lopalco PL. Tdap vaccination during pregnancy to protect newborns from pertussis infection. Ann Ig. 2018 Jul-Aug;30(4):346-363. doi: 10.7416/ai.2018.2226.

247 Pichichero ME, DeTora LM, Johnson D. An adolescent and adult formulation combined tetanus, diphtheria and five-component pertussis vaccine. Vaccine 2006;5:175–187.

248 Pichichero ME, Blatter M, Kennedy WA, et al. Acellular pertussis vaccine booster combined with diphtheria and tetanus toxoids for adolescents. Pediatrics 2006;117:1084–1093.

249 Pichichero ME, Rennels MB, Edwards KM, et al. Combined tetanus, diphtheria, and 5-component pertussis vaccine for use in adolescents and adults. JAMA 2005;293:3003–3011.

250 Talbot EA, Brown KH, Kirkland KB, et al. The safety of immunizing with tetanus-diphtheria-acellular pertussis vaccine (Tdap) less than 2 years following previous tetanus vaccination: experience during a mass vaccination campaign of healthcare personnel during a respiratory illness outbreak. Vaccine 2010;28:8001–8007.

251 Klein NP, Hansen J, Lewis E, et al. Post-marketing safety evaluation of a tetanus toxoid, reduced diphtheria toxoid and 3-component acellular pertussis vaccine administered to a cohort of adolescents in a United States health maintenance organization. Pediatr Infect Dis J 2010;29:613–617.

252 Yih WK, Nordin JD, Kulldorff M, et al. An assessment of the safety of adolescent and adult tetanus-diphtheria-acellular pertussis (Tdap) vaccine, using active surveillance for adverse events in the Vaccine Safety Datalink. Vaccine 2009;27:4257–4262.

253 Halperin S. How soon after a prior tetanus-diphtheria vaccination can one give adult formulation tetanus-diphtheria-acellular pertussis vaccine? Pediatr Infect Dis 2006;25:195–200.

254 Koepke R, Eickhoff JC, Ayele RA, et al. Estimating the effectiveness of tetanus-diphtheria-acellular pertussis vaccine (Tdap) for preventing pertussis: evidence of rapidly waning immunity and difference in effectiveness by Tdap brand. J Infect Dis. 2014;210:942-953.

255 Worby CJ, Kenyon C, Lynfield R, et al. Examining the role of different age groups, and of vaccination during the 2012 Minnesota pertussis outbreak. Sci Rep 2015;17:13182.

256 Witt MA, Arias L, Katz PH, et al. Reduced risk of pertussis among persons ever vaccinated with whole cell pertussis vaccine compared to recipients of acellular pertussis vaccines in a large US cohort. Clin Infect Dis 2013;56:1248-1254.

257 Australian Government Department of Health. National Notifiable Diseases Surveillance System: Notifications of a selected disease by age group, sex and year. Available at http://www9.health.gov.au/cda/source/rpt_5_sel.cfm. Accessed April 25, 2020.

258 Sheridan SL, McCall BJ, Davis CA, et al. Acellular pertussis vaccine effectiveness for children during the 2009-2010 pertussis epidemic in Queensland. Med J Aust 2014;200:334-338.

259 Sheridan SL, Ware RS, Grimwood K, et al. Number and order of whole cell pertussis vaccines in infancy and disease protection. JAMA 2012;308:454-456.

260 Centers for Disease Control and Prevention. Pertussis epidemic Washington, 2012. MMWR Morbid Mortal Wkly Rep 2012;61:517-522.

261 State of Wisconsin, Department of Health Services. Annual Summary of Reported Pertussis, 2012. Available from: https://www.dhs.wisconsin.gov/immunization/2012asrpertussis.pdf. Accessed April 25, 2020.

262 Centers for Disease Control and Prevention. Pertussis cases by year (1922-2013). Available from: www.cdc.gov/pertussis/surv-reporting/cases-by-year.html. Access date, October 2015.

263 Klein NP, Bartlett J, Rowhani-Rahbar A, et al. Waning protection after fifth dose of acellular pertussis vaccine in children. N Engl J Med 2012;367:1012-1019.

264 Baxter R, Bartlett J, Rowhani-Rahbar A, et al. Effectiveness of pertussis vaccines for adolescents and adults: case-control study. BMJ 2013;347:f4249

265 Decker MD, Hosbach P, Johnson DR, et al. Estimating the effectiveness of tetanus-diphtheria-acellular pertussis vaccine. J Infect Dis 2015;211:497-498.

266 Sanstead E, Kenyon C, Rowley S, et al. Understanding trends in pertussis incidence: an agent-based model approach. Am J Public Health 2015;105:e42-47.

267 Kiraly N, Dharmage SC, Allen KJ. Reduced risk of pertussis among persons ever vaccinated with whole-cell pertussis vaccine compared to recipients of acellular pertussis vaccines may have been confounded by age. Clin Infect Dis 2013;57:770.

268 André P, Johnson DR, Greenberg DP, et al. Reduced risk of pertussis in whole-cell compared to acellular vaccine recipients is not supported when data are stratified by age. Clin Infect Dis 2013;57:1658-1660.

269 Klein NP, Bartlett J, Fireman B, et al. Comparative effectiveness of acellular versus whole-cell pertussis vaccines in teenagers. Pediatrics 2013;131:e1716-1722.

270 Sheridan SL, Frith K, Snelling TL, et al. Waning vaccine immunity in teenagers primed with whole cell and acellular pertussis vaccine: recent epidemiology. Expert Rev Vaccines 2014;13:1081-1106.

271 Gambhir M1, Clark TA2, Cauchemez S, et al. A change in vaccine efficacy and duration of protection explains recent rises in pertussis incidence in the United States. PLoS Comput Biol 2015;11:e1004138.

272 Ntezayabo B, De Serres G, Duval B. pertussis resurgence in Canada largely caused by a cohort effect. Pediatr Infect Dis J 2003;22:22-27.

273 Rendi-Wagner P, Kundi M, Mikolasek A, Vécsei A, Frühwirth M, Kollaritsch H. Hospital-based active surveillance of childhood pertussis in Austria from 1996 to 2003: estimates of incidence and vaccine effectiveness of whole-cell and acellular vaccine. Vaccine 2006;24:5960-5.

274 Zerbo O, Bartlett J, Goddard K, Fireman B, Lewis E, Klein NP. Acellular pertussis vaccine effectiveness over time. Pediatrics 2019;144. pii: e20183466. doi: 10.1542/peds.2018-3466. Epub 2019 Jun 10.

275 Mahmud AS, Lipsitch M, Goldstein E. On the role of different age groups during pertussis epidemics in California, 2010 and 2014. Epidemiol Infect 2019;147:e184. doi: 10.1017/S0950268819000761.

276 Brummelman J, Wilk MM, Han WG, et al. Roads to the development of improved pertussis vaccines paved by immunology. FEMS Pathogens and Disease 2015;73:ftv067.

277 Ross PJ, Sutton CE, Higgins S, et al. Relative contribution of Th1 and Th17 cells in adaptive immunity to Bordetella pertussis: towards the rational design of an improved acellular pertussis vaccine. PLoS Pathog 2013;9:e1003264.

278 da Silva Antunes R, Babor M, Carpenter C, et al. Th1/Th17 polarization persists following whole-cell pertussis vaccination despite repeated acellular boosters. J Clin Invest 2018;128:3853-3865. doi: 10.1172/JCI121309.

279 Plotkin SA. Composition of pertussis vaccine given to infants determines long-term T cell polarization. J Clin Invest 2018;128:3742-3744. doi: 10.1172/JCI122726.

280 Mills KH, Gerdts V. Mouse and pig models for studies of natural and vaccine-induced immunity to Bordetella pertussis. J Infect Dis 2014;209 Suppl 1:S16-9. doi: 10.1093/infdis/jit488.

281 Wilk MM, Borkner L, Misiak A, Curham L, Allen AC, Mills KHG. Immunization with whole cell but not acellular pertussis vaccines primes CD4 TRM cells that sustain protective immunity against nasal colonization with Bordetella pertussis. Emerg Microbes Infect 2019;8:169-185. doi: 10.1080/22221751.2018.1564630.

282 Solans L, Debrie AS, Borkner L, et al. IL-17-dependent SIgA-mediated protection against nasal Bordetella pertussis infection by live attenuated BPZE1 vaccine. Mucosal Immunol 2018;11:1753-1762. doi: 10.1038/s41385-018-0073-9.

283 Pinto MV, Merkel TJ. Pertussis disease and transmission and host responses: insights from the baboon model of pertussis. J Infect 2017;74 Suppl 1:S114-S119. doi: 10.1016/S0163-4453(17)30201-3.

284 Misegades LK, Winter K, Harriman K, et al. Association of childhood pertussis with receipt of 5 doses of pertussis vaccine by time since last vaccine dose, California, 2010. JAMA 2012;308:2126-2132.

285 Goldstein E, Worby CJ, Lipsitch M. On the role of different age groups and pertussis vaccines during the 2012 outbreak in Wisconsin. Open Forum Infect Dis 2018;5:ofy082. doi: 10.1093/ofid/ofy082.

286 World Health Organization. WHO recommendations for routine immunization - summary tables. Available at https://www.who.int/immunization/policy/immunization_tables/en/. Accessed April 25, 2020.

287 World Health Organization. Immunization, Vaccines and Biologicals: Data, statistics and graphics. Available at https://apps.who.int/immunization_monitoring/surveillance/data/en. Accessed April 25, 2020.

288 Liang JL, Tiwari T, Moro P, et al. Prevention of pertussis, tetanus, and diphtheria with vaccines in the United States: recommendations of the Advisory Committee on Immunization Practices (ACIP). MMWR Recomm Rep 2018; 67:1–44.

289 Tessier E, Campbell H, Ribiero S, et al. Impact of extending the timing of maternal pertussis vaccination on hospitalized infant pertussis in England, 2014 - 2018. Clin Infect Dis 2020, in press.

290 Hoffman J. How anti-vaccine sentiment took hold in the United States. The New York Times. 2019 Sep 23. Available at https://www.nytimes.com/2019/09/23/health/anti-vaccination-movement-us.html. Accessed April 22, 2020.

291 Godlee F, Smith J, Marcovitch H. Wakefield's article linking MMR vaccine and autism was fraudulent. BMJ 2011;342:c7452. doi: 10.1136/bmj.c7452.

292 Phadke VK, Bednarczyk RA, Salmon DA, Omer SB. Association Between Vaccine Refusal and Vaccine-Preventable Diseases in the United States: A Review of Measles and Pertussis. JAMA 2016;315):1149-58. doi: 10.1001/jama.2016.1353

293 Glanz JM, McClure DL, Magid DJ, Daley MF, France EK, Salmon DA, Hambidge SJ. Parental refusal of pertussis vaccination is associated with an increased risk of pertussis infection in children. Pediatrics 2009;123:1446-51. doi: 10.1542/peds.2008-2150.

294 Glanz JM, Narwaney KJ, Newcomer SR, et al. Association between undervaccination with diphtheria, tetanus toxoids, and acellular pertussis (DTaP) vaccine and risk of pertussis infection in children 3 to 36 months of age. JAMA Pediatr 2013;167:1060-4. doi: 10.1001/jamapediatrics.2013.2353.

295 Gershon AA, Edwards K, Orenstein W, Schaffner W. Freedom, measles, and freedom from measles. N Engl J Med 2020;382:983-985. doi: 10.1056/NEJMp2000807.

296 Locht C, Mielcarek N. Live attenuated vaccines against pertussis. Expert Rev Vaccines 2014;13:1147-1158.

297 Mielcarek N, Debrie AS, Raze D, et al. Live attenuated B. pertussis as a single-dose nasal vaccine against whooping cough. PLoS Pathog 2006;2:e65.

298 Feunou PF, Kammoun H, Debrie AS, et al. Long-term immunity against pertussis induced by a single nasal administration of live attenuated B. pertussis BPZE1. Vaccine 2010;28:7047-7053.

299 Kammoun H, Feunou PF, Foligne B, et al. Dual mechanism of protection by live attenuated Bordetella pertussis BPZE1 against Bordetella bronchiseptica in mice. Vaccine 2012;30:5864– 5870.

300 Skerry CM, Mahon BP. A live, attenuated Bordetella pertussis vaccine provides long-term protection against virulent challenge in a murine model. Clin Vaccine Immunol 2011;18:187-193.

301 Feunou PF, Kammoun H, Debrie AS, et al. Heterologous prime-boost immunization with live attenuated B. pertussis BPZE1 followed by acellular pertussis vaccine in mice. Vaccine 2014;32:4281-4288.

302 Jahnmatz M, Amu S, Ljungman M, et al. B-cell responses after intranasal vaccination with the novel attenuated Bordetella pertussis vaccine strain BPZE1 in a randomized phase I clinical trial. Vaccine 2014;32:3350-3356.

303 US National Library of Medicine. ClinicalTrials.gov. Study of BPZE1 (High Dose) nasal live attenuated B. pertussis vaccine. Available at https://clinicaltrials.gov/ct2/results?term= NCT02453048&Search=Search. Accessed April 25, 2020.

304 Allen AC, Wilk MM, Misiak A, Borkner L, Murphy D, Mills KHG. Sustained protective immunity against Bordetella pertussis nasal colonization by intranasal immunization with a vaccine-adjuvant combination that induces IL-17-secreting TRM cells. Mucosal Immunol 2018;11:1763-1776. doi: 10.1038/s41385-018-0080-x.

305 Locht C, Papin JF, Lecher S, et al. Live attenuated pertussis vaccine BPZE1 protects baboons against Bordetella pertussis disease and infection. J Infect Dis 2017;216:117-124. doi: 10.1093/infdis/jix254.

306 Debrie AS, Coutte L, Raze D, et al. Construction and evaluation of Bordetella pertussis live attenuated vaccine strain BPZE1 producing Fim3. Vaccine 2018;36:1345-1352. doi: 10.1016/j.vaccine.2018.02.017.

307 Debrie AS, Mielcarek N, Lecher S, Roux X, Sirard JC, Locht C. Early Protection against Pertussis Induced by Live Attenuated Bordetella pertussis BPZE1 Depends on TLR4. J Immunol 2019;203:3293-3300. doi: 10.4049/jimmunol.1901102.

308 Lin A, Apostolovic D, Jahnmatz M, et al. Live attenuated pertussis vaccine BPZE1 induces a broad antibody response in humans. J Clin Invest 2020 Mar 23. pii: 135020. doi: 10.1172/JCI135020.

309 Thorstensson R, Trollfors B, Al-Tawil N, et al. A phase I clinical study of a live attenuated Bordetella pertussis vaccine--BPZE1; a single centre, double-blind, placebo-controlled, dose-escalating study of BPZE1 given intranasally to healthy adult male volunteers. PLoS One 2014;9:e83449. doi: 10.1371/journal.pone.008344.

310 Locht C, Mielcarek N. New pertussis vaccination approaches: en route to protect newborns? FEMS Immunol Med Microbiol 2012;66:121–133,.

311 Gracia A, Polewicz M, Halperin SA, et al. Antibody responses in adult and neonatal BALB/c mice to immunization with novel Bordetella pertussis vaccine formulations. Vaccine 2011;29:1595–1604.

312 Asokanathan C, Corbel M, Xing D. A CpG-containing oligodeoxynucleotide adjuvant for acellular pertussis vaccine improves the protective response against Bordetella pertussis. Hum Vaccin Immunother 2013;9:325-331.

313 Sharma S, Benson HA, Mukkur TK, et al. Preliminary studies on the development of IgA-loaded chitosan-dextran sulphate nanoparticles as a potential nasal delivery system for protein antigens. J Microencapsul 2013;30:283-294.

314 Alvarez Hayes J, Erben E, Lamberti Y, et al. Identification of a new protective antigen of Bordetella pertussis. Vaccine 2011;29:8731-8739.

315 Alvarez Hayes J, Erben E, Lamberti Y, et al. Bordetella pertussis iron regulated proteins as potential vaccine components. Vaccine 2013;31:3543-3548.

316 Cheung GY, Xing D, Prior S, et al. Effect of different forms of adenylate cyclase toxin of Bordetella pertussis on protection afforded by an acellular pertussis vaccine in a murine model. Infect Immun 2006;74:6797-6805.

317 Asensio CJ, Gaillard ME, et al. Outer membrane vesicles obtained from Bordetella pertussis Tohama expressing the lipid A deacylase PagL as a novel acellular vaccine candidate. Vaccine 2011;29:1649-1656.

318 Roberts R, Moreno G, Bottero D, et al. Outer membrane vesicles as acellular vaccine against pertussis. Vaccine 2008;26:4639-4646.

319 de Gouw D, Serra DO, de Jonge MI, et al. The vaccine potential of Bordetella pertussis biofilm-derived membrane proteins. Emerg Microbes Infect 2014;3:e58.

320 Robbins JB, Schneerson R, Kubler-Kielb J, et al. Toward a new vaccine for pertussis. Proc Natl Acad Sci 2014;111:3213-3216.

321 Buasri W, Impoolsup A, Boonchird C, et al. Construction of Bordetella pertussis strains with enhanced production of genetically-inactivated Pertussis Toxin and Pertactin by unmarked allelic exchange. BMC Microbiol 2012;12:61.

322 Hozbor DF. Outer membrane vesicles: an attractive candidate for pertussis vaccines. Expert Rev Vaccines 2017;16:193-196. doi: 10.1080/14760584.2017.1276832.

323 Zurita ME, Wilk MM, Carriquiriborde F, et al. A pertussis outer membrane vesicle-based vaccine induces lung-resident memory CD4 T cells and protection against Bordetella pertussis, including pertactin deficient strains. Front Cell Infect Microbiol 2019;9:125. doi: 10.3389/fcimb.2019.00125.

324 Dorji D, Mooi F, Yantorno O, Deora R, Graham RM, Mukkur TK. Bordetella Pertussis virulence factors in the continuing evolution of whooping cough vaccines for improved performance. Med Microbiol Immunol 2018;207:3-26. doi: 10.1007/s00430-017-0524-z.

325 Boehm DT, Hall JM, Wong TY, et al. Evaluation of adenylate cyclase toxoid antigen in acellular pertussis vaccines by using a Bordetella pertussis challenge model in mice. Infect Immun 2018;86. pii: e00857-17. doi: 10.1128/IAI.00857-17.

326 Nguyen AW, Wagner EK, Laber JR, et al. A cocktail of humanized anti-pertussis toxin antibodies limits disease in murine and baboon models of whooping cough. Sci Transl Med 2015;7:316ra195. doi: 10.1126/scitranslmed.aad0966.

327 Garlapati S, Eng NF, Kiros TG et al. Immunization with PCEP microparticles containing pertussis toxoid, CpG ODN and a synthetic innate defense regulator peptide induces protective immunity against pertussis. Vaccine 2011;29:6540–6548.

328 Allen AC, Mills KH. Improved pertussis vaccines based on adjuvants that induce cell-mediated immunity. Expert Rev Vaccines 2014;13:1253-1264.

329 Agnolon V, Bruno C, Leuzzi R, et al. The potential of adjuvants to improve immune responses against TdaP vaccines: A preclinical evaluation of MF59 and monophosphoryl lipid A. Int J Pharm 2015;492:169-176.

330 Misiak A, Leuzzi R, Allen AC, et al. Addition of a TLR7 agonist to an acellular pertussis vaccine enhances Th1 and Th17 responses and protective immunity in a mouse model. Vaccine 2017;35:5256-5263. doi: 10.1016/j.vaccine.2017.08.009.

331 Jennings-Gee J, Quataert S, Ganguly T, D'Agostino R Jr, Deora R, Dubey P. The adjuvant Bordetella Colonization Factor A attenuates alum-induced Th2 responses and

enhances Bordetella pertussis clearance from mouse lungs. Infect Immun 2018;86. pii: e00935-17. doi: 10.1128/IAI.00935-17.

332 Atwell JE, Van Otterloo J, Zipprich J, et al. Nonmedical vaccine exemptions and pertussis in California, 2010. Pediatrics 2013;132:624-630.
